# Life histories as mosaics: Plastic and genetic components differ among traits that underpin life‐history strategies

**DOI:** 10.1111/evo.14440

**Published:** 2022-02-06

**Authors:** Anja Felmy, David N. Reznick, Joseph Travis, Tomos Potter, Tim Coulson

**Affiliations:** ^1^ Department of Zoology University of Oxford Oxford OX1 3SZ United Kingdom; ^2^ Department of Evolution, Ecology and Organismal Biology University of California, Riverside Riverside California 92521; ^3^ Department of Biological Science Florida State University Tallahassee Florida 32306

**Keywords:** Co‐gradient variation and counter‐gradient variation, common garden experiment, life‐history evolution, phenotypic plasticity, reaction norm, resource dependence

## Abstract

Life‐history phenotypes emerge from clusters of traits that are the product of genes and phenotypic plasticity. If the impact of the environment differs substantially between traits, then life histories might not evolve as a cohesive whole. We quantified the sensitivity of components of the life history to food availability, a key environmental difference in the habitat occupied by contrasting ecotypes, for 36 traits in fast‐ and slow‐reproducing Trinidadian guppies. Our dataset included six putatively independent origins of the slow‐reproducing, derived ecotype. Traits varied substantially in plastic and genetic control. Twelve traits were influenced only by food availability (body lengths, body weights), five only by genetic differentiation (interbirth intervals, offspring sizes), 10 by both (litter sizes, reproductive timing), and nine by neither (fat contents, reproductive allotment). Ecotype‐by‐food interactions were negligible. The response to low food was aligned with the genetic difference between high‐ and low‐food environments, suggesting that plasticity was adaptive. The heterogeneity among traits in environmental sensitivity and genetic differentiation reveals that the components of the life history may not evolve in concert. Ecotypes may instead represent mosaics of trait groups that differ in their rate of evolution.

Life‐history strategies of populations often differ consistently between habitats. The life histories and phenotypes associated with specific habitats are known as “ecotypes” and may evolve repeatedly in parallel. Examples include the variation in shell size and growth rate among ecotypes of the marine snail *Littorina saxatilis* (Butlin et al. 2014), the accelerated maturation and smaller adult body size of the “dwarf” compared to the “normal” ecotypes of Lake whitefish (Rogers and Bernatchez 2007), and the slow‐living “meadow” and fast‐living “lakeshore” ecotypes of the western terrestrial garter snake (Bronikowski and Arnold 1999).

Life‐history differences between ecotypes can result from phenotypic plasticity, genetic differentiation, and genotype‐by‐environment interactions (Stearns 1992). The magnitude and direction of components of variation have important consequences for trait evolution and population dynamics, and potentially for species’ adaptive potential and resilience to environmental change (Robinson and Dukas 1999; West‐Eberhard 2003; Gienapp et al. 2008; Merilä and Hendry 2014). For example, when plasticity brings the mean phenotype closer to the optimum, as is often assumed to happen when plastic and genetic differences are the same sign (so‐called co‐gradient variation), mean fitness is increased, the selection differential reduced, and adaptive change proceeds more slowly (Price et al. 2003; Coulson et al. 2017). By contrast, when plasticity moves the mean phenotype further from the optimum, as when plastic and genetic responses have different signs (counter‐gradient variation), the selection differential is increased and adaptive change proceeds more rapidly (Ghalambor et al. 2007; Coulson et al. 2021).

Ecotypes emerge from multidimensional clusters of traits. For example, surface‐ and cave‐dwelling Atlantic mollies differ in a variety of life‐history traits, including body length, fat content, reproductive investment, fecundity, and offspring size (Riesch et al. 2010, 2011). Life‐history differences between ecotypes are multilayered in numerous species, often coupled with behavioral, morphological, and physiological divergence (e.g., Bronikowski and Arnold 1999; Rogers and Bernatchez 2007; Matesanz et al. 2020). It is tempting to describe the life‐history variation between two ecotypes along a single axis of overall variation along which there are trade‐offs between opposing values of many individual traits, for example, a fast versus slow pace of life (Promislow and Harvey 1990; Blackburn 1991) or *r*‐ versus *K*‐selection (Macarthur and Wilson 1967; Pianka 1970). Although heuristically useful, this simplification risks underestimating the complexity of life‐history evolution and overlooking two interesting questions.

The first question, common in the literature, is how genetic covariances among traits at the onset of local adaptation either facilitate or hinder the rate of divergence between ecotypes (Lande and Arnold 1983; Arnold 1992; Schluter 1996; Steppan et al. 2002). If genetic covariances among the individual traits that define ecotypes are aligned with the multivariate direction of selection, then the clusters will appear quickly and divergence occur rapidly. If genetic covariances are largely orthogonal to the direction of multivariate selection, then divergence will require mutations, or processes that reduce linkage disequilibrium, to change the alignment of genetic covariances (Falconer and Mackay 1996), and divergence will take a longer and perhaps more labored pathway.

The second question, which has received less attention, is whether the traits that cluster into ecotypes respond to environmental variation in a uniform manner and with uniform magnitude. If they do, then all traits will have a similar partitioning of phenotypic variation into genetic and environmental components and display similar directional responses to the same environmental gradients. The rate of multivariate evolution will then largely be guided by genetic covariances. If, however, this is not the case, some traits would display more environmental variation than others and would respond in opposite directions than others to the same environmental gradient. The rates of evolution of the traits in these clusters could then differ substantially, making the construction of ecotypes a much slower process. Additionally, different responses to the same environmental gradient would make correlations between traits highly variable among environmental conditions (e.g., Simons and Roff 1996; Travis et al. 1999; Niva and Jokela 2000). Those correlations determine the gradients of direct and indirect selection (Roff 1997; Coulson et al. 2018); if they vary with environment, then the evolution of ecotypes is further slowed down, and ecotypes themselves represent mosaics of trait groups, rather than simple clusters.

The ecotypes of Trinidadian guppies (*Poecilia reticulata*), a species of small, live‐bearing freshwater fish, represent an ideal system to estimate among‐trait variation in plastic and genetic components of life‐history strategies. Their divergent life histories are not only a well‐studied example of rapid, repeatable evolutionary change in the wild (Reznick 1982; Reznick and Endler 1982; Reznick and Bryga 1996; Reznick et al. 1997, 2019), we also have a good understanding of the key environmental drivers in this system. Guppy ecotypes are adapted to different sections of natural streams in the Northern Range Mountains of Trinidad. Guppies in downstream river sections experience high per‐capita food levels (Grether et al. 2001; Zandonà et al. 2017), but population densities are kept low by intense predation (Gilliam et al. 1993; Reznick et al. 2001). By contrast, in upstream habitats (above waterfalls that act as barriers to large piscivorous fishes), guppies experience low per‐capita food levels. In the absence of intense predation, guppies exist at much higher densities in these localities, thereby reducing food availability.

Guppy ecotypes have distinct life histories consistent with selection for either fast or slow reproduction (characteristics of ecotypes and their habitats summarised in Table 1; Reznick 1982; Reznick and Bryga 1996). In the guppy literature, these ecotypes are usually referred to as “high‐ versus low‐predation” ecotypes. For clarity, we will here call them “fast‐ versus slow‐reproducing” ecotypes, acknowledging that this dichotomy is oversimplistic. In downstream habitats, guppies tend to have younger ages and smaller sizes at maturity, produce more, but smaller, offspring per litter, have shorter interbirth intervals, and invest more resources in reproduction than in upstream habitats (Reznick and Endler 1982; Reznick and Bryga 1987; Reznick et al. 1990, 1996). The repeated evolution of the slower life‐history strategy from fast‐reproducing ancestors occurs primarily in response to increased population density, rather than as a direct effect of reduced predation risk (Bassar et al. 2013; Reznick et al. 2019; Reznick and Travis 2019) or reduced per‐capita food availability (Reznick 1982; Reznick and Bryga 1996; Reznick et al. 2019). However, differences in the *realized* life histories of natural guppy populations will not only reflect genetic differences due to density‐dependent selection, but also plastic effects of different per‐capita food levels in up‐ and downstream habitats. Whether genetic and plastic effects differ among the traits that define these ecotypes, and thus whether the ecotypes represent a single cluster of traits or a mosaic of groups of traits, remains unknown.

**Table 1:** Characteristics of guppy ecotypes in Trinidad

	Fast‐reproducing ecotype	Slow‐reproducing ecotype	References
Ecological variables:			
Predation intensity	Higher	Lower	Gilliam et al. 1993
Population density	Lower	Higher	Reznick et al. 2001
Primary productivity	Higher	Lower	Grether et al. 2001
Invertebrate biomass	Higher	Lower	Zandonà et al. 2017
Phenotypic variables:			
Age at maturity	Younger	Older	Reznick 1982; Reznick and Bryga 1987, 1996; Reznick et al. 1990, 2019
Size at maturity	Smaller	Larger	Reznick and Endler 1982; Reznick and Bryga 1987; Reznick et al. 1990, 1996, 2019; Grether et al. 2001; Zandonà et al. 2011; Potter et al. 2021
Litter size	Larger	Smaller	Reznick 1982; Reznick et al. 1990, 1996, 2001, 2004; Reznick and Bryga 1987, 1996; Zandonà et al. 2011
Offspring size	Smaller	Larger	Reznick 1982; Reznick and Endler 1982; Reznick and Bryga 1987, 1996; Reznick et al. 1990, 1996, 2001
Inter‐birth interval	Shorter	Longer	Reznick 1982; Reznick and Endler 1982; Reznick and Bryga 1996; Reznick et al. 2006
Investment in reproduction	Higher	Lower	Reznick 1982; Reznick and Endler 1982; Reznick et al. 1990, 1996, 2001; Reznick and Bryga 1996

Here, we report a test of this idea using the divergent life‐history strategies of fast‐ and slow‐reproducing guppies and a laboratory common‐garden approach that manipulates food availability, a key feature of habitat contrasts between ecotypes. To account for the complexity of life histories, we included 36 traits underlying life‐history variation in guppies, encompassing attributes of both parents and offspring at multiple breeding attempts. For each of these traits, we evaluated the extent to which differences between guppy ecotypes are due to phenotypic plasticity in response to food level, genetic differences between ecotypes, or ecotype‐by‐food‐level interactions. We then compared the relative directions of genetic and plastic changes, to assess whether plasticity was more likely to facilitate or constrain evolutionary change in each trait. As life‐history traits may often be nonindependent (Van Noordwijk and De Jong 1986; Pigliucci 2003), we also estimated their phenotypic correlation matrix. We tested four hypotheses (listed in Table 2 along with expected results), focused, in turn, on plastic effects, genetic effects, their relative directions, and their interactions.

**Table 2:** The hypotheses and expected results of this study

Hypothesis	Expected result
1. Most life‐history traits respond plastically to variation in food availability.	Significant effect of experimental food level on more than half of the studied traits.
2. Plastic traits are genetically differentiated between ecotypes, whereas nonplastic traits lack genetic effects.	Lack or low frequency of traits with plastic but no genetic effects, or genetic but no plastic effects.
3. Where both plastic and genetic effects exist, their directions are aligned (i.e., co‐gradient variation).	Same‐sign changes from the higher to the lower food level as from the fast‐ to the slow‐reproducing (and, in the field, food‐limited) ecotype in traits where both plastic and genetic effects are significant.
4. The sensitivity to food scarcity does not differ between ecotypes.	Nonsignificant ecotype‐by‐food‐level interaction.

We expected food effects to be ubiquitous (hypothesis 1) because life‐history traits are emergent properties of how the whole phenotype interacts with the environment (Coulson et al. 2006), an important aspect of which is resource availability. Trait expression will depend on the resources individuals can accrue and on how these are allocated to potentially competing fitness components (Van Noordwijk and De Jong 1986; Coulson 2020).

There are several reasons why traits’ plastic and genetic components ought to be correlated (hypothesis 2; Hansen et al. 2011). Both environmental and genetic variation is expected to increase with a trait's complexity: a composite, polygenic trait with intricate development should be more sensitive to both environmental and genetic perturbations than a trait whose genetic architecture is simple (Houle 1992; Hansen et al. 2011). Moreover, stabilizing selection should reduce both plastic and genetic components of variation (Houle 1992; Hansen et al. 2011).

For traits with both plastic and genetic differences between ecotypes, we expected them to take the same sign (hypothesis 3). This is based on the assumption that plastic responses to differential per‐capita food availability may have preceded, or co‐occurred with, the evolution of same‐sign genetic changes between guppy ecotypes. Specifically, we expected the plastic effect of the lower food level to be aligned with the genetic effect of the slow‐reproducing ecotype.

Lastly, we tested for interactions between food levels and ecotypes (hypothesis 4). Such interactions could indicate that ecotypes are adapted to their local per‐capita food levels. Previous work has not detected them (Reznick 1982; Reznick and Bryga 1996; Reznick et al. 2019), but we aimed to confirm this here using a larger dataset.

## Materials and Methods

### STUDY SPECIES

Trinidadian guppies are small poeciliid fish native to freshwater streams on the island of Trinidad, West Indies. They display sexual dimorphism. Males are smaller than females as male growth is determinate, with virtually no further growth after sexual maturity has been attained (Reznick 1990). Females keep growing throughout most of their lives until eventually reaching an asymptotic body length (Reznick et al. 2006). Guppies are live‐bearing. Females reproduce for the first time at 60–140 days of age and give birth to subsequent litters of young at intervals of 22–35 days (Reznick et al. 2006). Litter size increases for 7–8 months after maturity, ranging from 3 to 45 offspring per litter, then levels off (Reznick et al. 2004). In captivity, females live for 11–44 months and produce an average of 15–28 litters (Reznick et al. 2006); in the field, mean life expectancy is substantially lower. Although guppies experience a seasonal environment, reproduction takes place all year round. Offspring are precocial and do not receive any parental care.

### OVERVIEW OF METHODS

We used four datasets from four independent experiments with identical design, all of them aimed at estimating the relative importance of environmental (i.e., food‐level) versus genetic (i.e., ecotype) effects on life‐history traits in Trinidadian guppies (details in Methods S1 and Table S1). Datasets 1, 2, and 4 have previously been published, whereas dataset 3 is published here for the first time. Our combined dataset is available from Dryad (Felmy et al. 2022). It encompasses guppies from seven fast‐reproducing and nine slow‐reproducing populations situated in a total of seven drainages (see map in Fig. S1). These drainages, separated by watershed divides or by the sea, represent at least six putatively independent evolutionary origins of the slow‐reproducing ecotype, as indicated by low degrees of genome‐wide relatedness of guppies inhabiting different drainages (Willing et al. 2010; Fraser et al. 2015; Whiting et al. 2021). This experimental design gives us ample power to assess which life‐history traits show patterns with respect to food and ecotype that are general across drainages (i.e., parallel evolution), or unique to individual drainages (i.e., genetic divergence). Altogether, our data include two within‐river paired comparisons of both ecotypes from the south slope and five from the north slope of the Northern Range Mountains of Trinidad. Localities were considered to be inhabited by fast‐ or slow‐reproducing guppies depending on the presence or absence of major predators of guppies: the pike cichlid (*Crenicichla alta*) and the wolf fish (*Hoplias malabaricus*) on the south slope (Gilliam et al. 1993), and several species of gobies on the north slope (Reznick et al. 1996). We note, however, that the distribution of gobies in the north slope streams has not been well investigated, so the meaning of high versus low predation might not correspond one to one in the north and south slope streams.

Each dataset consists of a factorial experiment in the laboratory, in which two levels of a daily food ration (high vs. low) were crossed with the two ecotypes (fast‐ vs. slow‐reproducing). Males were measured for five and females for 31 life‐history traits. Details of the laboratory rearing protocol and of the measurement of traits can be found in Methods S2 and S3. All measured traits are also briefly described in Table 3. By using second‐generation, laboratory‐reared fish derived from field‐caught females, keeping fish in a common environment on controlled amounts of food, and splitting pairs of full‐siblings between food levels, we eliminated maternal, environmental, and other nonheritable sources of variation to the greatest extent. Any trait differences between ecotypes therefore indicate genetic differentiation. Effects of food levels are evidence of phenotypic plasticity, and differences between ecotypes in their response to food levels imply genotype‐by‐environment interactions.

**Table 3:** Traits considered in this study and details of how they were modeled

Trait information					Modeling details			
Description	Abbreviation	Sex	Unit	Stage	Transformation	Maternal identity	Drainage	Covariates
Standard length (tip of snout to hypural plate in tail)	len1‐len3	f	mm	1,2,3	untr/log/sqrt	R/R/R	R/R/R	–/–/–
	lenmat	m	mm	mat	log	R	R	–
Dry weight of reproductive tissues	repwt	f	mg	3	sqrt	R	F	–
Dry weight of somatic tissues	somwt	f	mg	3	log	R	F	–
Litter size (# offspring per brood)	n1‐n3	f	#	1,2,3	log/sqrt/sqrt	R/R/R	R/R/R	–/–/–
Maternal‐size‐adjusted litter size	n1_wt1adj‐n3_wt3adj	f	#	1,2,3	log/sqrt/sqrt	R/R/R	R/R/R	wt1/wt2/wt3
Wet weight	wt0‐wt3	f	mg	0,1,2,3	log/log/log/log	R/R/R/R	R/R/R/R	–/–/–/–
	wt0m, wtmat	m	mg	0, mat	log/log	R/R	R/R	–/–
Interbirth interval (# days between consecutive broods)	intrvl1, intrvl2	f	d	2,3	log/log	R/R	R/R	–/–
Mean dry weight of offspring	mnemb1‐3	f	mg	1,2,3	untr/untr/untr	R/R/R	R/R/F	–/–/–
Age	age0, agepart1‐3	f	d	0,1,2,3	untr/log/log/log	–/R/R/R	R/R/R/R	–/–/–/–
	age0m, agemat	m	d	0, mat	untr/log	–/R	R/R	–/–
% fat of total dry weight	fat	f	%	3	untr	R	F	–
% fat of dry weight of reprod. tissues	repfat	f	%	3	log	R	F	–
% fat of dry weight of somatic tissues	somfat	f	%	3	untr	R	F	–
% fat of mean offspring dry weight	mnembfat1‐3	f	%	1,2,3	untr/sqrt/sqrt	R/R/R	R/R/F	–/–/–
Reproductive allotment (% dry weight of pregnant female that are offspring)	repall	f	%	3	untr	R	F	

Traits were measured at distinct stages: for females, these were the first, second, and third time they gave birth to a litter of young (stages 1–3); for males, it was the attainment of sexual maturity (mat); for both sexes, traits were also measured at the beginning of the controlled food treatment (stage 0). Traits that were measured more than once were considered unique traits and were analyzed separately. The weight and percentage fat of offspring were measured at birth. We modeled each trait individually, using appropriate data transformations and error structures to meet the assumptions of the linear mixed models. As fixed effects, all models included the food level (high and low), ecotype (fast‐ and slow‐reproducing), and dataset (1–4). Some models additionally included maternal weight as a covariate. Maternal identity and drainage were included as random intercepts (or, for traits with <5 drainages, as fixed effects), to account for nonindependence of data due to relatedness or common evolutionary history, respectively. f = female; m = male; mm = millimeters; mg = milligrams; # = number; d = days; % = proportion; untr = untransformed trait values; log = base‐*e* log‐transformed trait values; sqrt = square‐root transformed trait values; R = random effect; F = fixed effect.

### STATISTICAL ANALYSIS

#### Overview of analyses

We conducted analyses of datasets on their own and in combination. Because datasets used different daily rations of food, we first analyzed growth rates to assess the degree of differences in food levels among datasets. We then tested for differences between experimental food levels (i.e., phenotypic plasticity) and between ecotypes (i.e., genetic differentiation) in life‐history traits by fitting linear mixed‐effects models for each trait. Finally, we assessed the phenotypic correlation structure among traits to explore patterns of nonindependence.

We used a Bonferroni‐corrected significance level to control for an elevated rate of type I errors due to multiple testing (Bonferroni 1936). There are 36 measured traits, three tests for each trait (food‐level effects, ecotype effects, their interaction), and four datasets, amounting to 36 × 3 × 4 = 432 individual tests. Our critical value is therefore 0.05 / 432 = 0.0001157, or, reasonably, 0.0001. In all linear models, we looked for influential data points using function “CookD” in R‐package “predictmeans” (Luo et al. 2018), which calculates Cook's distance for each data point based on its leverage and residual value (Cook 1977). However, all data points lay inside 0.5 Cook's distance (maximum Cook's distance: 0.20) and thus have little apparent influence on the fitted values (Kutner et al. 2005), and excluding the few less‐extreme outliers that were present did not noticeably change our results. We thus retained all data in our final analyses.

Statistical analyses were performed in R version 3.4.0 (R Core Team 2017) and 4.0.0 (R Core Team 2020). The network plots of phenotypic correlations was prepared using R‐package “igraph” (Csardi and Nepusz 2006). Values are given as mean ± SD.

#### Differences in food levels between datasets

We used analysis of variance with repeated measures to test for differences in female growth rates as a function of the experimental food level, the dataset, the ecotype, and the drainage (details in Methods S4). The primary goal was to compare the chosen food levels among datasets by using growth as a proxy for the size of food rations. We focused on females because dataset 4 did not include males, and because females were weighed four times and males only twice.

These analyses showed that absolute food levels differed between datasets 1–4, yet the observed patterns of female growth between the start of experimental food treatments and the birth of litter 3 were repeatable (Results S1; Fig. S2; Tables S2 and S3). Hence, effects of food limitation were profound and did not depend on the exact food rations used.

#### Phenotypic plasticity and genetic differentiation

When analyzing the datasets jointly, we fitted a separate linear mixed‐effects model for each of the 36 traits to investigate effects of experimental food levels and ecotypes (fixed effects), while controlling for differences between datasets (fixed effect), drainages (fixed or random effect), and maternal identities (random effect). Table 3 provides information on the traits that were studied and on how they were modeled. As the reference level for categorical predictors, modeled using a dummy contrast coding scheme, we chose the higher food level, the fast‐reproducing ecotype, and dataset 2, as only dataset 2 contained all 36 traits. Details of the model selection process can be found in Methods S5. All models were fitted using R‐packages “lme4” (Bates et al. 2015) and “lmerTest” (Kuznetsova et al. 2017). We tested the significance of random effects by means of log‐likelihood ratio tests comparing the full model to one without the random effect in question. After selecting the best‐fitting model for each measured trait, we evaluated the plausibility of the model results by comparing them to plots of trait values against all predictors, separately for models with and without two‐way interactions.

For each trait, we obtained mean‐standardized effect sizes for experimental food levels and ecotypes from the best‐fitting model. These were computed by dividing the model estimate (i.e., the partial regression coefficient) for the effect of food or ecotype, respectively, by the intercept, and multiplying this ratio by 100.

In addition to analyses using the combined datasets, we conducted dataset‐specific analyses. For each dataset and trait present within the dataset, a separate model was fitted, resulting in a total of 112 models (details in Methods S6).

#### Phenotypic correlations

We assessed the phenotypic correlation structure between traits by computing Pearson product‐moment correlation coefficients between pairs of traits within each combination of food level and ecotype (main manuscript), and when pooling fish from all treatment combinations (Supporting Information). Correlations that persist within treatment combinations are not caused by shared sensitivity to food availability, nor by shared differentiation between up‐ and downstream habitats. We estimated correlations separately for traits measured in males and females.

## Results

### PLASTICITY WITH RESPECT TO FOOD AVAILABILITY

**Figure 1:**
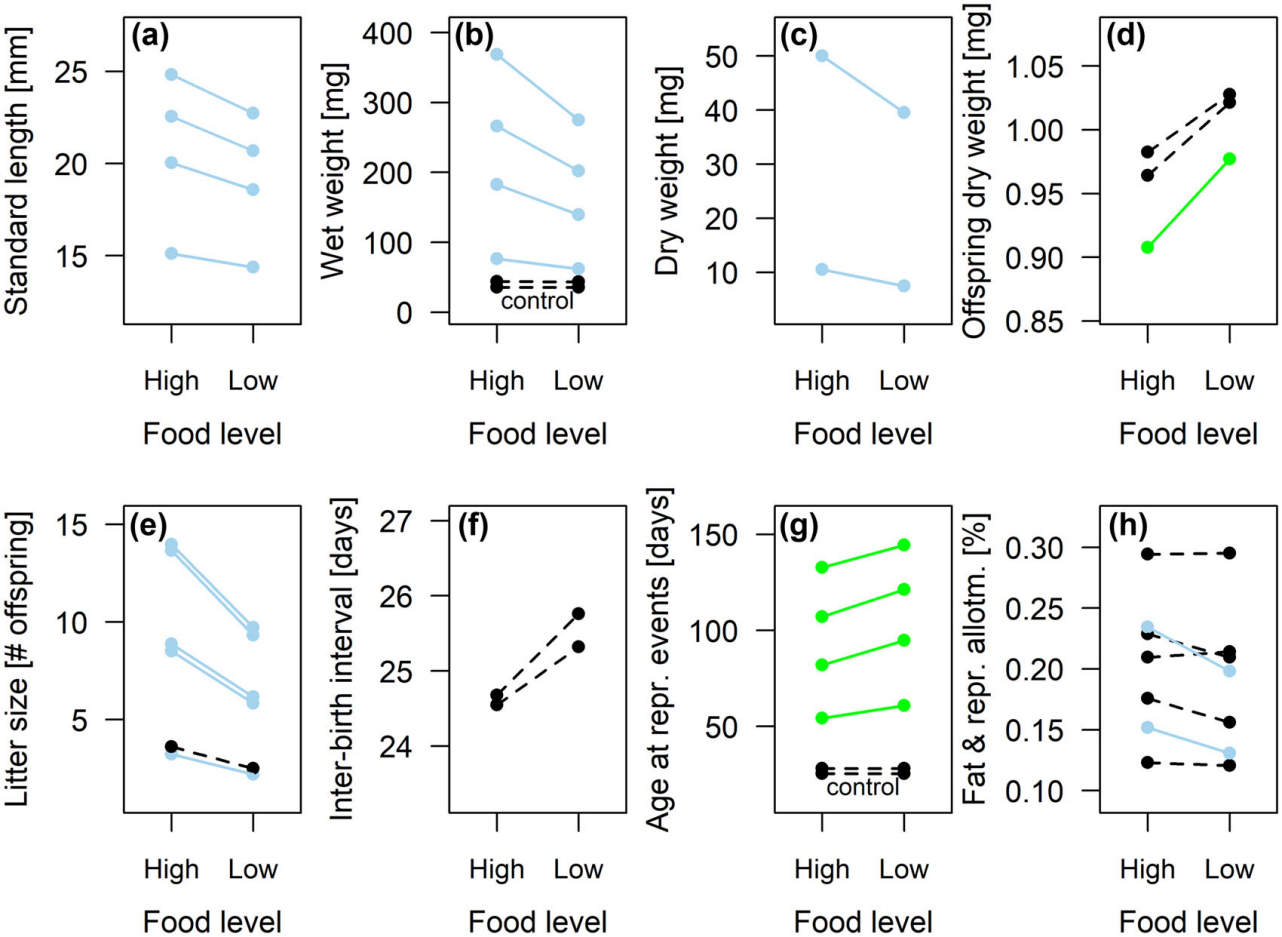
Support for hypothesis 1: The majority of 36 life‐history traits were plastic with respect to food availability. Reaction norms show predicted mean values at the higher and lower food level, with significant decreases in blue and significant increases in green. Nonsignificant changes are shown as dashed black lines. Effects were considered significant when *P* < 0.0001 (i.e., ∼0.05/432), following Bonferroni (1936), to account for an increased type I error rate due to multiple testing. A separate model was fitted for each of 36 traits (details in Table 3). Models included the experimental food level (high vs. low), the ecotype (fast‐ vs. slow‐reproducing), and the dataset (1–4) as categorical fixed effects, with a reference level of high food, in the fast‐reproducing ecotype, using dataset 2. Models of size‐adjusted litter size (E) include postpartum maternal weight as a covariate. Random effects were maternal identity nested within drainage, unless there were only four drainages, in which case drainage was fitted as a categorical fixed effect. For full model results, see Tables S4–S39. Four traits were measured before controlled food treatments began and thus serve as negative controls for food effects. In panels B, E, and G, a small amount was added to some values to increase the visibility of nearby data points.

Twenty‐two out of 32 life‐history traits measured after the start of food treatments (68.8%) showed significant phenotypic plasticity in response to food levels (Fig. 1; full model results in Tables S4–S39). At the lower food level, fish were consistently shorter (Fig. 1a), lighter (Figs. 1b, c), and older when they reproduced (Fig. 1g) than at the higher food level. For example, under low‐food conditions females were, on average, 7.3% shorter, 23.7% lighter (wet weight), and 13.5% older at first birth, whereas males were 4.9% shorter, 18.9% lighter, and 10.7% older when attaining sexual maturity (averages based on predicted means for food levels when pooling ecotypes). Food limitation also increased offspring size in a female's first litter by 7.1% on average (Fig. 1d), while reducing the number of offspring in all three litters by nearly one third (31.5–31.7% reduction; Fig. 1e). Finally, low food levels lowered the percentage fat in females’ somatic tissues by 13.8% and the percentage fat in offspring belonging to litter 3 by 15.5% (Fig. 1h).

Only 10 traits (31.3%) did not respond with significant plasticity to food levels. These were both interbirth intervals (Fig. 1f), offspring size in litters 2 and 3 (Fig. 1d), offspring number in litter 1 when adjusting for maternal weight (Fig. 1e), the reproductive allotment, the percentage fat in females’ total and reproductive tissues, and the percentage fat in offspring belonging to litters 1 and 2 (all Fig. 1h). Additionally, the weight and age of both sexes at the initiation of controlled feeding regimes, included as control traits, showed no effects of food levels (Fig. 1b, g).

As more than half of the studied traits, and traits as biologically distinct as weights, ages, and litter sizes, were significantly affected by food levels, we concluded that our data support hypothesis 1, which posited that most life‐history traits of Trinidadian guppies are plastic with respect to food availability.

### GENETIC DIFFERENTIATION BETWEEN ECOTYPES

**Figure 2:**
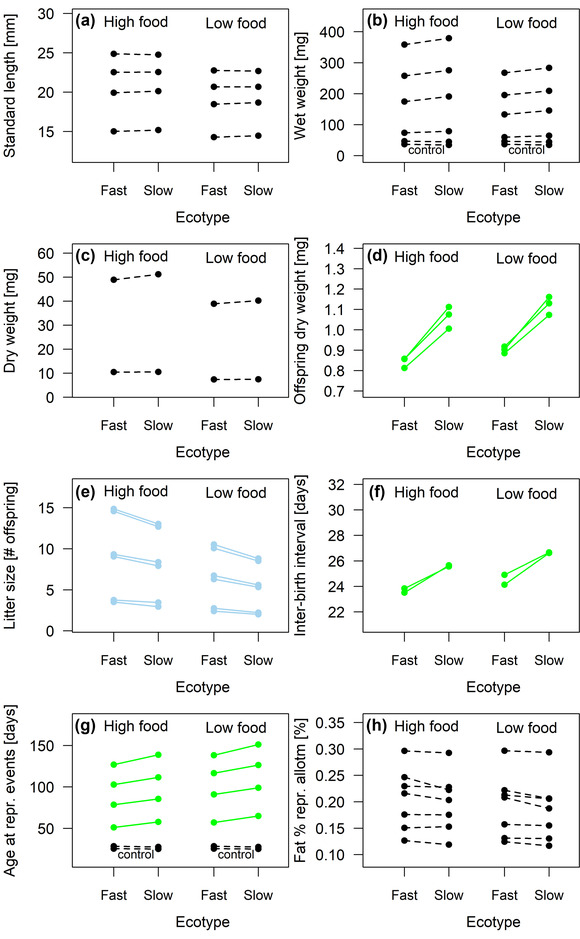
No support for hypothesis 2: Phenotypic plasticity, or its lack, does not predict genetic differentiation between ecotypes. Reaction norms show predicted mean values for the fast‐ and slow‐reproducing ecotype, separately for fish kept at the higher and lower food level. Twelve traits with significant plasticity (Fig. 1) lacked significant genetic effects, whereas five nonplastic traits were significantly differentiated between ecotypes. Significant decreases are shown as blue lines, significant increases as green lines, and nonsignificant changes as dashed black lines. Effects were considered significant when *P* < 0.0001 (i.e., ∼0.05/432), following Bonferroni (1936), to account for an increased type I error rate due to multiple testing. Four traits were measured at the beginning of experiments in all fish, and so serve as negative controls for ecotype effects. In panels B, E, and G, a small amount was added to some values to increase the visibility of nearby data points.

Fifteen out of 32 traits (46.9%) showed significant genetic differences between ecotypes (Fig. 2; full model results in Tables S4‐S39). Fish from the slow‐reproducing ecotype had a longer developmental period from juvenile to adult (Fig. 2g), longer intervals between consecutive births (Fig. 2f), and produced smaller litters (Fig. 2e) of larger offspring (Fig. 2d) than fish from the fast‐reproducing ecotype. On average, female age at first birth was delayed by 8.8% in the slow‐reproducing ecotype, with knock‐on delays in female age at second (8.4%) and third birth (9.2%). Male age at maturity was delayed by 13.2% (Fig. 2g, averages based on predicted means for ecotypes when pooling food levels). Interbirth intervals were 7.1–9.7% longer (Fig. 2f). Litters contained between 13.6% and 16.0% fewer offspring (Fig. 2e), but these were substantially larger, with an increase in newborn dry weights of 22.5% in the first, 24.2% in the second, and 29.2% in the third litter (Fig. 2d).

For 17 traits measured after experiments had begun (53.1%) and four control traits measured before, ecotypes were not detectably different. These included all size‐related traits (adult lengths, adult wet and dry weights; Fig. 2a, b, c), percentages of fat in both adults and offspring, the reproductive allotment (Fig. 2h), and male and female age and weight at the beginning of experiments (control traits; Fig. 2b, g).

Contrary to expectations, traits that responded plastically to food levels were not more likely to be genetically differentiated between ecotypes. Of the 22 traits with significant plasticity, only 10 (45.5%) had significant genetic effects, whereas of the 10 nonplastic traits, only five (50.0%) also lacked significant differences between ecotypes. In other words, 17 traits (53.1%) had either plastic but no genetic effects, or genetic but no plastic effects—two categories whose expected frequencies were low under hypothesis 2, which predicted that traits’ environmental and genetic variances are correlated. Consequently, hypothesis 2 was not supported by our data.

### DIRECTIONS OF PLASTIC AND GENETIC CHANGES

**Figure 3:**
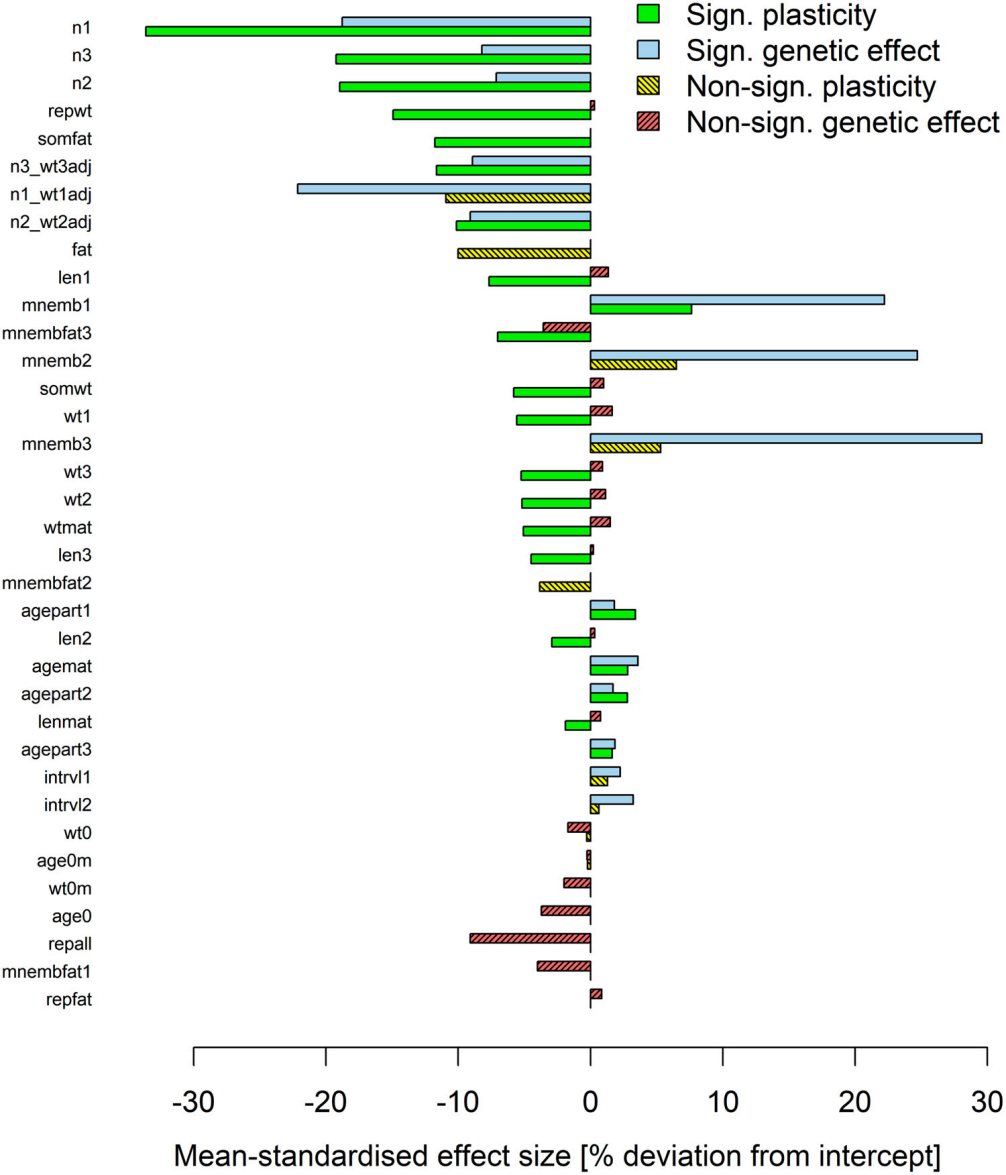
Support for hypothesis 3: Where both plastic and genetic effects existed, their directions were aligned, indicating that these traits showed co‐gradient plasticity. Shown are mean‐standardized effect sizes of experimental food levels (i.e., phenotypic plasticity) and ecotypes (i.e., genetic differentiation). Food‐level effects show changes from the higher to the lower food level among fish of both ecotypes, whereas ecotype effects show changes from the fast‐ to the slow‐reproducing ecotype among fish of both food levels. Without exception, traits for which both plastic and genetic changes were significant exhibited co‐gradient plasticity, as they showed consistent effects of the lower food level and of originating from the slow‐reproducing (food‐limited) ecotype. Effects were considered significant when *P* < 0.0001 (i.e., ∼0.05/432), following Bonferroni (1936), to account for an increased type I error rate due to multiple testing. For traits that were transformed before models were fitted, results are provided on the transformed scale. Traits were ordered by decreasing absolute amount of plasticity. Trait abbreviations are explained in Table 3.

Traits differed substantially in the magnitude of plastic and genetic changes: when significant, plasticity accounted for a change in trait means of 8.6% ± 7.6% (range: 1.6–33.6%), and genetic predisposition for a change of 11.0% ± 9.7% (range: 1.7–29.6%; Fig. 3). Where both changes were significant, these pointed in the same direction (Fig. 3). Traits’ effect sizes for food levels and ecotypes were either both positive or both negative, indicating that comparing a higher to a lower food level was qualitatively similar to comparing the fast‐reproducing ecotype (which inhabits high‐food environments) to the slow‐reproducing ecotype (which typically is food‐limited). For traits in which the lower food level caused a decrease or increase in trait values, so did belonging to the slow‐reproducing ecotype (Fig. 3). Not a single trait had significant plastic and genetic effects of different signs (i.e., showed counter‐gradient plasticity).

Accordingly, we concluded that traits with both plastic and genetic components exhibited co‐gradient plasticity. Our data thus supported hypothesis 3, which postulated that the plastic and genetic effects of life‐history traits in Trinidadian guppies are aligned.

### INTERACTIONS BETWEEN FOOD LEVELS AND ECOTYPES

There was very little evidence suggesting that the fast‐reproducing ecotype, which in the field experiences higher per‐capita food levels, was more sensitive to food scarcity than the slow‐reproducing ecotype. The interaction between food levels and ecotypes was not detectable for all traits (31 traits *P* ≥ 0.05, five traits 0.05 > *P* ≥ 0.0026). The lack of genotype‐by‐environment interactions is illustrated in Figure 2, which shows that food levels barely affected differences between ecotypes (i.e., reaction norms of the ecotypes to food levels are parallel). Neither could we detect ecotype‐by‐food‐level interactions in our analyses of female growth rates, conducted to assess variation in food levels between datasets (*F*_1_ ≤ 2.60, *P* ≥ 0.11; Tables S2,S3); these results remained unchanged when ecotype was fitted as the first predictor in analyses of variance (*F*_1_ ≤ 3.1, *P* ≥ 0.08).

Accordingly, hypothesis 4, which predicted that plasticity to per‐capita food availability does not differ between ecotypes, was supported by our data, in agreement with previous work (Reznick 1982; Reznick and Bryga 1996; Reznick et al. 2019).

### DIFFERENCES BETWEEN DATASETS, DRAINAGES, AND MOTHERS

Dataset‐specific analyses for each trait showed that most effects of food levels and ecotypes were consistent across datasets. Although there were small differences in effect sizes for a few traits, effects found in the combined dataset were mirrored in individual datasets (data not shown). The same picture emerged from models of the combined dataset that included interactions between datasets and food levels, and between datasets and ecotypes (data not shown). This was despite considerable differences among datasets in mean trait values, which were most notable in strongly plastic traits (Tables S4–S39). For example, adult fish and litter sizes were significantly larger in the unpublished dataset 3, where food levels were highest, than in other datasets. It should be noted that datasets also differed in the drainages and localities sampled; however, as our models included drainage as an additional predictor (see below), we are confident that dataset effects mostly reflect variation in food levels.

The drainage of origin of experimental fish significantly affected some traits (Tables S4–S39). For instance, in their first litter, females from the Yarra drainage were 6.3% shorter, 22.9% lighter, and produced 20.9% smaller embryos than females from the Oropuche drainage (averages based on raw data), despite being part of the same dataset and hence fed identical food quantities. In analyses when drainage was treated as a random effect (28 traits), drainage accounted for 12.9% ± 8.3% (range: 0.0–34.8%) of the variance explained by random effects. Importantly, the magnitude of ecotypic differences and the effects of food‐level variation also varied among drainages (see Figs. S3–S9 for seven traits showing drainage‐specific effects). For example, for female age at first birth, the effect of food‐level variation was dramatic in the El Cedro but minimal in the Marianne. Conversely, ecotypic differences were small in the El Cedro but quite large in the Yarra.

Maternal identity explained variation in male length, weight, and age at sexual maturity, and in the weight of both sexes at the onset of experimental treatments (Tables S4–S39). The influence of maternal identity on other traits was mixed, with some traits affected by it (e.g., interbirth interval 2) but closely related ones not (e.g., interbirth interval 1). Note that maternal identity is confounded with variation in the laboratory microenvironment, as maternal siblings were housed in adjacent tanks.

### SYNTHESIS AND PHENOTYPIC CORRELATION STRUCTURE

**Figure 4:**
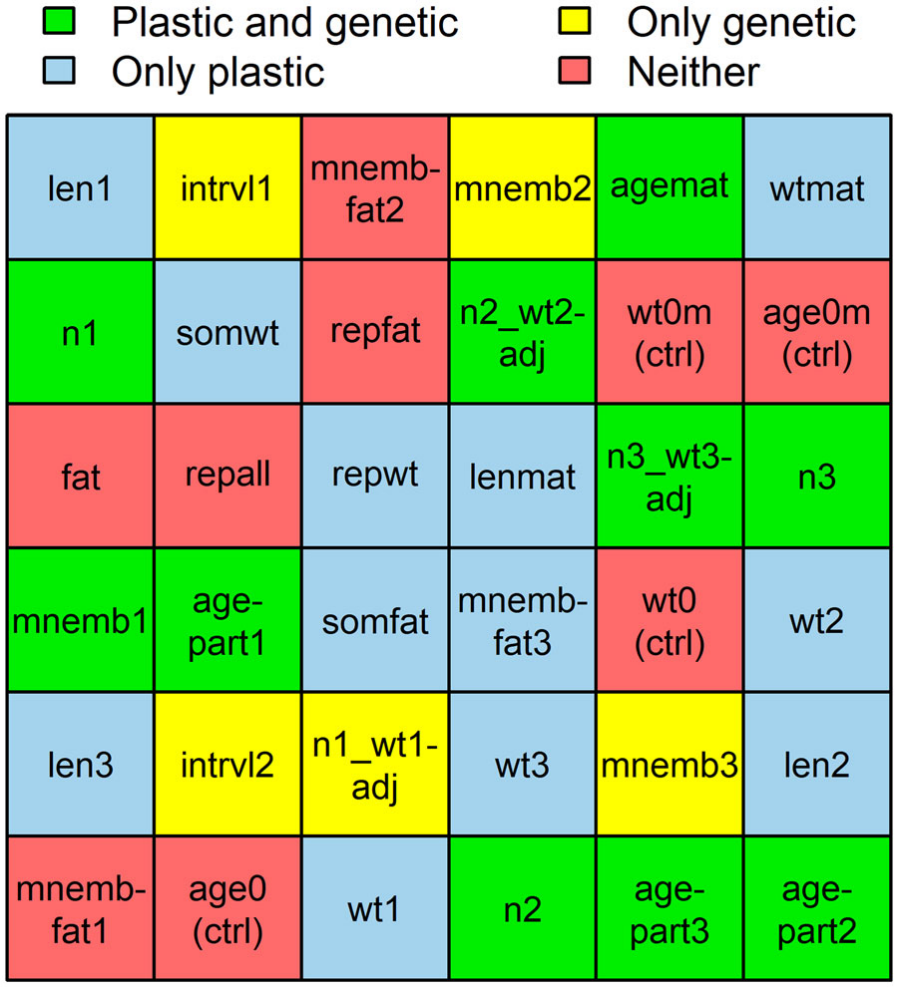
Life histories as mosaics: Plastic and genetic components differed among traits that, together, form the fast‐ and slow‐reproducing guppy ecotypes. Traits could be assigned to four groups based on the presence or absence of significant phenotypic plasticity with respect to food levels and significant genetic differentiation between ecotypes (indicated with colors). The position of traits within the mosaic is random. Trait abbreviations are explained in Table 3.

Taken together, we found that the divergent life histories of Trinidadian guppies emerge from a mosaic of traits with unequal levels of environmental and genetic control (Fig. 4). Ten traits, including reproductive scheduling and most litter sizes, had both plastic and genetic components. Twelve traits, including all size‐related traits, were primarily plastic. Five traits, including interbirth intervals and most offspring sizes, showed mainly genetic variation. And nine traits, including the percentage fat, the reproductive allotment, and four control traits, had neither detectable plastic nor detectable genetic components. Where both plastic and genetic changes existed, they pointed in the same direction, indicating that these traits exhibited co‐gradient plasticity. The degree of plasticity did not differ between ecotypes, seeing as interactions between food levels and ecotypes were nondetectable. Results were consistent across datasets but differed, for some traits, substantially among drainages.

**Figure 5:**
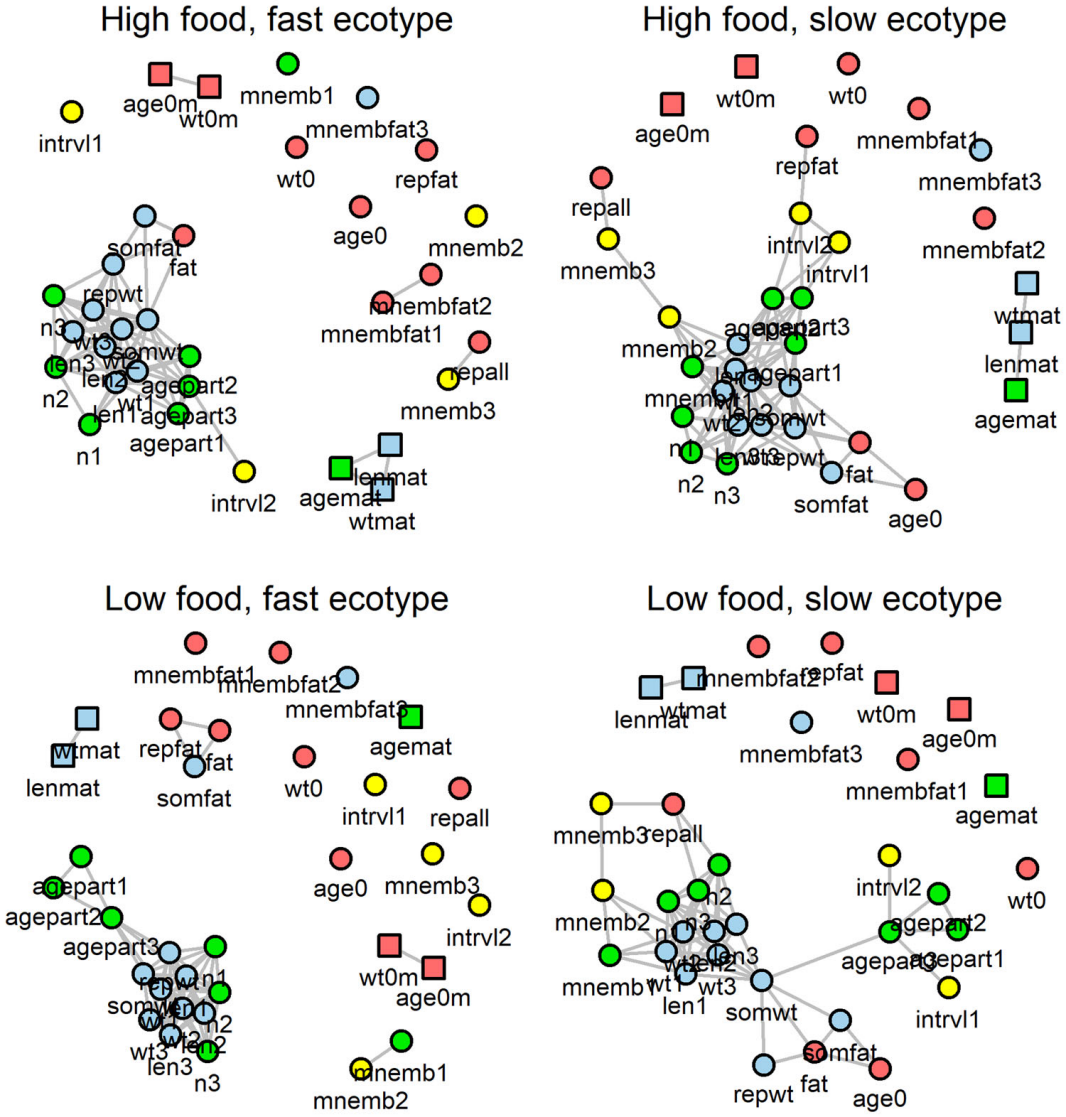
Phenotypic correlations were strongest between traits with similar plastic and genetic components. Correlations were computed separately for the four combinations of food levels and ecotypes. The coloring indicates whether traits had significant plastic and genetic components (green), only plastic components (blue), only genetic components (yellow), or neither (red). Gray lines connect traits with pairwise Pearson product‐moment correlation coefficients of *r* ≥ 0.5. The correlation between traits measured in females (circles) and males (squares) is *r* = 0 by definition. The shorter the connecting line between two traits, the stronger is their correlation. The position of traits and trait clusters relative to one another is irrelevant. Trait abbreviations are explained in Table 3.

A number of traits in these analyses were correlated with one another (Fig. 5). Three features of the correlation structure of the data support the argument that life histories evolve as mosaics. First, within each combination of food level and ecotype, there is a mixture of correlated and uncorrelated traits. Each group contains a cluster of highly correlated traits and a scattering of many uncorrelated traits; the latter often lacked both plastic and genetic effects. Second, the correlation structure among traits is generally consistent within each combination of food level and ecotype, so does not simply reflect shared responses to the experimental treatments. For example, the reproductive allotment of females and fat content of embryos were always independent of nearly all other variables. At the other extreme, the ages and sizes of females at successive births were always highly correlated with one another. Third, the strongest correlations were among traits with similar plastic and genetic components (Figs. 4 and 5). For example, traits describing female size, which differed between food levels but not ecotypes, were strongly correlated with one another (*r* = 0.90 ± 0.06), as were the ages of females at consecutive births, which differed between both food levels and ecotypes (*r* = 0.92 ± 0.07). Correlations between traits with dissimilar plastic and genetic components (i.e., different colors in Fig. 5) were typically weaker (e.g., between female weights/lengths and litter sizes: *r* = 0.58 ± 0.09, between female weights/lengths and female ages: *r* = 0.37 ± 0.19). The same patterns appeared when correlations were computed across treatment combinations (Fig. S10).

The strong correlation structure means that some traits studied here are not fully independent of one another, potentially reducing statistical power for testing hypotheses 1 and 2. However, for each treatment combination, significant plasticity was present among members of several clusters, as well as in uncorrelated traits (Fig. 5), showing that guppy life histories indeed strongly depended on food availability (hypothesis 1). Similarly, traits with components contradicting hypothesis 2 (i.e., either plastic only or genetic only) were distributed across clusters (Fig. 5). The lack of support for hypothesis 2, indicating that plasticity did not necessarily co‐occur with genetic differentiation, was thus not caused by phenotypic correlations.

## Discussion

The fast and slow life histories of Trinidadian guppies inhabiting high‐ and low‐predation habitats are well‐documented (Reznick and Endler 1982; Reznick and Bryga 1987; Reznick et al. 1990, 1996). Here, we used this study system to examine whether the individual traits that make up a life‐history strategy have similar levels of environmental and genetic control. We found that they do not: traits varied substantially in how food‐level‐induced plasticity and genetic disposition contributed to the divergence of ecotypes. Although some traits had both plastic and genetic components (litter size, reproductive timing), others exhibited only plasticity (body size), only genetic differentiation (inter‐birth intervals, offspring size), or neither (percentage fat, reproductive allotment). Biologically related traits, such as consecutive weight measurements, had similar components; this was true also for statistically independent traits (e.g., offspring weights in successive litters). Moreover, components were similar between the male and female counterparts of traits, despite, by design, nonexistent phenotypic correlations between traits measured in separate sexes. Within sexes, phenotypic correlations were stronger between traits with similar and weaker between traits with dissimilar components.

Few studies of ecotypic variation have combined common garden experiments with manipulations of specific environmental factors to estimate plastic and genetic components of life‐history traits. In the small shrub *Lepidium subulatum*, flowering onset, inflorescence number, and reproductive biomass showed both genetic differences between populations and plasticity in response to water stress, whereas fruiting onset had only genetic effects, flower number and inflorescence size only plastic effects, and seed size neither (Matesanz et al. 2020). In the surface‐ and cave‐dwelling ecotypes of Atlantic mollies, both male and female life‐history traits showed all possible combinations of genetic components and plastic reactions to food levels and lighting conditions (Riesch et al. 2016). Similar results were found with respect to temperature for urban and rural grasshoppers (San Martin y Gomez and Van Dyck 2012) and for meadow and lakeshore western terrestrial garter snakes (Bronikowski 2000). These studies, mirroring our own findings, thus tentatively suggest that heterogeneity among traits in levels of plasticity and genetic differentiation might be common.

The presence of variation among traits in environmental and genetic components is not a statistical inevitability of using a 2 × 2 factorial design. It would have been entirely possible to find that some of the four combinations of plastic and genetic effects were not represented among the traits studied here. For example, traits without significant plasticity could also have lacked significant genetic differentiation, and vice versa (as assumed by hypothesis 2), leaving two of the four categories empty. Our data show that this was not the case: 17 traits showed significant effects of either food levels or ecotypes, but not both, whereas 10 traits showed effects of both, and nine (five when excluding control traits) effects of neither. At this point, we do not know why we failed to find the expected positive correlation between environmental and genetic variation (Houle 1992; Hansen et al. 2011); potential reasons include, but are not limited to, ostensibly nonplastic traits exhibiting plasticity to environmental variables other than food abundance, or a trait's lack of overall genetic differentiation resulting from differences among drainages in the direction of ecotypic divergence (see below). Moreover, we could have (but have not) found that some traits with both plastic and genetic components exhibited counter‐gradient variation, effectively creating a fifth category. The lack of such traits is further evidence for the biological validity, rather than statistical inevitability, of our results.

### LIFE HISTORIES AS MOSAICS: PREDICTIONS OF UNEQUAL RATES OF EVOLUTION

The variation in the magnitude and direction of traits’ plastic and genetic components almost certainly has consequences for life‐history evolution. Based on our findings, we can make cautious predictions of the likely rate of phenotypic and evolutionary change in different groups of traits. We assume a scenario in which guppies from one ecotype are transplanted to, or naturally invade, localities typical for the other ecotype. Consequently, the main selective forces would be competition for food in upstream and predator avoidance in downstream habitats. A scenario of reciprocal invasion is plausible seeing as guppies are very successful invaders, having become established after introductions to every continent except Antarctica (Deacon et al. 2011). Our tentative predictions are as follows:

First, we expect that predominantly plastic traits, such as adult body size, might quickly change phenotypically as trait expression shifts within a single generation to match the new food environment (Woltereck 1909; Lewontin 1974; Kawecki and Ebert 2004). The rate of genetic change in primarily plastic traits is more difficult to predict because it depends on the amount of additive genetic variance these traits possess within ecotypes, something that our study did not estimate. Note that the absence of significant ecotype effects does not mean that, within ecotypes, individual differences in traits do not have a genetic basis. Second, primarily genetic traits, such as interbirth intervals and offspring sizes, might have a slower rate of phenotypic change, as change can only result from change in allele frequencies (assuming that traits are not plastic to other aspects of the environment). Accordingly, rates of genetic and phenotypic change should be similar. Third, when traits had both plastic and genetic components, as was the case for litter size and the age at which individuals reproduced, these components were aligned: the lower food level caused traits to change in the same direction as did belonging to the slow‐reproducing (and, in the field, food‐limited) ecotype. Such co‐gradient variation is often assumed to indicate that plasticity is adaptive (Price et al. 2003; Coulson et al. 2017). Traits like these might quickly change phenotypically because of their plasticity (Conover and Schultz 1995; Robinson and Dukas 1999; West‐Eberhard 2003; Levis and Pfennig 2016), but evolution may proceed rather slowly because plasticity will bring phenotypes closer to local optima for all genotypes equally, thereby increasing mean fitness but masking advantageous genotypes from selection and reducing the selection differential (Ghalambor et al. 2007; Coulson et al. 2017). Finally, neither phenotypic nor evolutionary change is expected to happen in traits without plasticity nor genetic differentiation.

This diversity of expected rates of change suggests that the life‐history strategies of Trinidadian guppies are mosaics of traits that can evolve and respond to environmental variation in contrasting ways, and yet form the ecotypes that are so readily apparent. To broadly speak of “life‐history evolution” thus likely masks the complex interplay of genes and environment on the multiple traits that underpin life‐history strategies.

At this time, our predictions must be considered speculative. For one thing, they assume that food availability is a major environmental driver of realized life‐history differences between guppy ecotypes in the field; the persistent absence of significant interactions between experimental food level and ecotype argues against food availability as an agent of selection. In truth, life‐history traits could be plastic with respect to other aspects of the environment, such as social density, predation risk, or parasite pressure. For another thing, our predictions assume that the magnitude of differences in laboratory food levels is comparable to or at least as great as natural variation in per‐capita food levels. Although our results were consistent across four datasets with unequal food levels, demonstrating their robustness to variation in food quantity, the diets of laboratory fish differed from those of free‐living fish: we fed our fish high‐protein liver paste and brine shrimp nauplii, rather than the low‐calorie algae typically present at upstream sites (Grether et al. 2001; Zandonà et al. 2017). If the slow‐reproducing ecotype was better at scraping off and metabolizing low‐quality periphyton, our study would be underestimating the effects of differences in food levels. Neither did we measure competitive ability. By housing fish individually, we tested whether slow‐reproducing fish can better tolerate a low‐quantity diet, which they could not, given the general lack of interactions between food levels and ecotypes. Yet, the slow‐reproducing ecotype may be adapted to food scarcity in ways we did not measure, such as by feeding more quickly or being a superior competitor to the fast‐reproducing ecotype.

### PARALLELISM VERSUS DIVERGENCE IN LIFE‐HISTORY EVOLUTION

For some traits, patterns of food and habitat dependence were consistent across the seven drainages included in our study, whereas for others they were not. Where patterns were consistent, they provide strong evidence for parallel evolution (see also Reznick and Bryga 1996; Reznick et al. 1996) and suggest that natural selection acts similarly in similar environments (Muir 1924). However, many traits showed differences among drainages in mean trait values, in the amount of plasticity, and, interestingly, in the extent of genetic differentiation. In some traits, such as female length at first birth and male length at maturity, there was substantial variation among drainages in the magnitude of ecotypic differences (Figs. S3–S9).

Spatial variation in the strength of ecotypic parallelism and divergence is more common than long assumed (Bolnick et al. 2018) and has been found in many systems, such as in sticklebacks (Ravinet et al. 2013; Liu et al. 2018), lake whitefish (Landry et al. 2007), flat periwinkles (Galindo et al. 2021), and yellow monkeyflowers (Lowry et al. 2008). Such variation could have multiple causes (Bolnick et al. 2018), of which we will discuss three. First, selection pressures may differ between independent origins of ecotypes, in response to more fine‐grained environmental heterogeneity. For example, the degree of parallelism in ecotypic divergence in lake whitefish is influenced by the extent of hypolimnetic oxygen depletion (Landry et al. 2007). In Trinidad, canopy openness, algal standing crops, and predator communities differ not only between the upstream and downstream sites of a given drainage, but also between drainages (Grether et al. 2001; Reznick et al. 2001) and between the north and south slope of the Northern Range Mountains (Reznick et al. 1996). Accordingly, fine‐scale selection regimes experienced by guppies likely differ among drainages. Indeed, a previously detected nonparallelism in guppy life histories was associated with increased mortality due to disease and flooding (Fitzpatrick et al. 2014).

Second, differences in time since divergence are likely to affect the magnitude of ecotypic differentiation (Berner et al. 2010; Lucek et al. 2014). In our system, drainages vary in the history of their populations, with upstream populations in some drainages being more recently derived from downstream populations than others. Such differences should show up as variation among drainages in the amount of within‐stream migration and in estimated divergence times between up‐ and downstream populations. Genetic analyses have confirmed these a posteriori hypotheses (Willing et al. 2010; Fraser et al. 2015; Blondel et al. 2019). In particular, ecotypic effects were limited for several female traits measured in dataset 1, which used fish from a 4‐year introduction experiment (Reznick and Bryga 1987).

**Figure 6:**
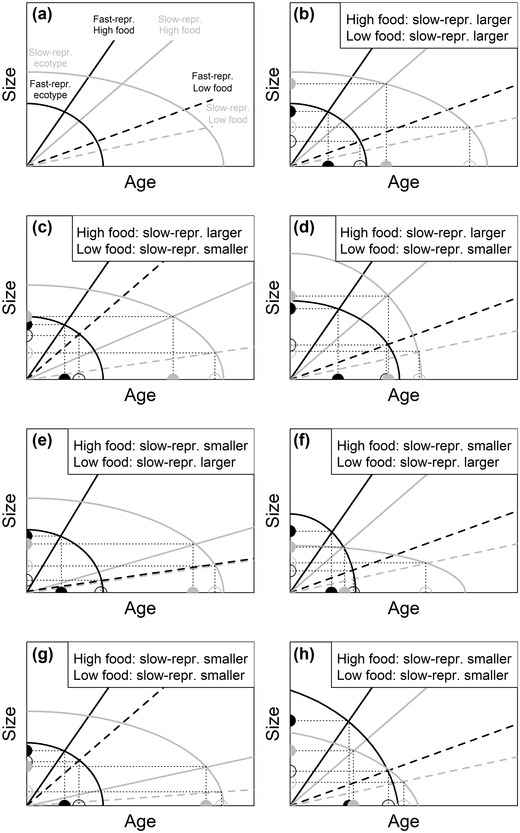
Theoretical prediction of changes in body size because of variation in growth rates (a, c, e, g) and in threshold rules for maturation (b, d, f, h). Male size at sexual maturity and female size at first birth are determined by the intersection of growth rates (straight lines) with threshold rules defining the age and size at reproductive events (curved lines). Fish from the fast‐reproducing (black lines) and slow‐reproducing ecotype (gray lines) potentially differ in threshold rules for maturation, and in how they grow innately or in response to high (solid lines) and low food availability (dashed lines). Shown is how size at maturity/first birth reacts to changes in growth rates while keeping threshold rules constant (a, c, e, g), and to changes in threshold rules while keeping growth rates constant (b, d, f, h). Note that change in either one alone is sufficient to produce all possible ways in which sizes can differ between ecotypes at high and low food availability. (a, b) Same initial situation, with size at maturity/first birth larger in the slow‐reproducing ecotype irrespective of food levels. (c, d) Size still larger in the slow‐reproducing ecotype under high food, but smaller under low food. (e, f) Reversed situation. (g, h) Size smaller in the slow‐reproducing ecotype at both food levels. In all scenarios, the age at maturity/first birth is younger in the fast‐reproducing ecotype, and younger under high food, in accordance with results found here and in previous studies.

Third, populations may have different genetic routes via which adaptation occurs (Kautt et al. 2014; Westram et al. 2014; Fang et al. 2020; Whiting et al. 2021). Genomic analyses showed that guppies in different drainages are highly divergent from one another (Willing et al. 2010), suggesting that the sort of genetic variation selection can act upon might differ among drainages. Let us consider adult body size, which, depending on the drainage, was larger, smaller, or identical in fast‐ and slow‐reproducing fish. Male size at maturity and female size at first birth are determined by the intersection of growth rates with threshold rules for maturation (Day and Rowe 2002; Nilsson‐Örtman and Rowe 2021). Threshold rules define the age and size at reproductive events (Fig. 6a, b). When assuming that both growth rates and threshold rules vary among drainages (shown for growth rates by Arendt and Reznick 2005; Potter et al. 2019), among up‐ and downstream habitats, and, certainly for growth rates, in response to food availability, it becomes clear how ecotype effects on adult size can vary across drainages. Every possible pattern of adult size with respect to ecotype and food can be produced by varying either the growth rates (Fig. 6a, c, e, g) or the threshold rules (Fig. 6b, d, f, h), demonstrating that identical phenotypes can be produced via different genetic routes, and different phenotypes via identical routes.

Among‐drainage variation in ecotype effects, driven by environmental (Grether et al. 2001; Reznick et al. 2001) and genetic (Willing et al. 2010; Fraser et al. 2015; Whiting et al. 2021) differences among drainages, could mask the overall effect of ecotype when measured across multiple drainages. This might have happened here with adult size‐related traits. Most previous studies, whether conducted in the field (Reznick and Endler 1982; Reznick and Bryga 1987; Reznick et al. 1990, 1996; Zandonà et al. 2011) or laboratory (Reznick 1982; Reznick and Bryga 1987, 1996; Reznick et al. 1990, 2019; Grether et al. 2001; Potter et al. 2021), found that adult lengths and weights of both sexes were significantly larger in the slow‐reproducing than in the fast‐reproducing ecotype. A second potential source of the difference in our conclusions is our stringent criterion for significance (*P* < 0.0001). For example, our data revealed an ecotype effect for female wet weight at first birth (*P* = 0.00037) that would be considered highly significant in conventional data interpretations.

### TRAITS WITHOUT PLASTICITY OR GENETIC DIFFERENTIATION

The prevalence of plasticity in response to food availability (seen in 69% of traits) emphasizes the pervasive effects of resources on life histories and corroborates our understanding of life histories as complex, emergent properties of interactions between the phenotype and its (resource) environment (Van Noordwijk and De Jong 1986; Coulson et al. 2006; Coulson 2020). This finding tallies with life‐history studies of food effects in other fishes (e.g., Riesch et al. 2016; Felmy et al. 2021). However, 10 traits measured after the start of food treatments were largely unaffected by food levels, including interbirth intervals, most offspring weights, the percentage fat in both females and offspring, and the reproductive allotment. Interestingly, food‐independent traits were only loosely embedded in the phenotypic correlation matrix. The absence of correlations eliminates potential indirect effects of food availability, further reducing the impact of resources on food‐insensitive traits.

It is worth noting that some of these traits are proportions (percentage fat, reproductive allotment). Food levels can have no effect on proportions in two broad ways: by truly affecting neither numerator nor denominator, or by affecting both proportionately so that the ratio itself stays constant. As some numerators (e.g., total dry weight of offspring in a female's last litter) and denominators (e.g., female dry weight, offspring dry weight in litter 1) of ostensibly food‐insensitive proportions were found to depend on food levels, our results do not necessarily contradict the notion that resources have near‐ubiquitous effects on life histories.

Every second nonplastic trait also lacked genetic differentiation; only interbirth intervals and offspring weights proved relatively unaffected by food levels yet differed among guppy ecotypes. Considering all the traits studied here, it appears that low resource availability impacts reproduction primarily by slowing down maturation from juvenile to adult, with at least medium‐term consequences for a female's entire reproductive schedule, and by reducing the number of offspring per litter. The maturation period in utero and the size of newborn offspring, however, seem mainly determined by genetic effects, with very little influence of resources (although perhaps of environmental aspects other than food availability).

For the remaining traits, the putative reasons for the lack of food and habitat effects are diverse. They range from females potentially compensating for low food levels by growing and maturing more slowly yet maintaining investment into fat reserves (Pugliese 1988; Auer 2010) to the coarse nature of the ether extractions performed to remove stored fat, which might have dissolved not only triglycerides, the main constituents of body fat, but other types of lipids too (Cowey and Sargent 1972).

## Conclusion

In this study, we have quantified the extent to which ecotypic variation in the life histories of Trinidadian guppies is due to phenotypic plasticity, genetic differentiation, and genotype‐by‐environment interactions. We have considered a large set of traits, to do justice to the multidimensionality of differences between ecotypes. Our results underscore this complexity by showing that life histories are composed of traits that differ in their contributions of plastic and genetic components, in agreement with studies in other systems (Bronikowski 2000; San Martin y Gomez and Van Dyck 2012; Riesch et al. 2016; Matesanz et al. 2020). The composite nature of life‐history strategies was further supported by the presence of stronger phenotypic correlations between traits with similar than between traits with dissimilar components. Future studies will need to show whether our finding of dissimilar variance partitioning across a multitude of life‐history traits is replicated across other systems. If it is, there might be an opportunity for reducing the number of traits under study, particularly if they are biologically distinct (e.g., weights, ages, litter sizes). However, such generality would also mean that anthropogenic change risks disrupting optimal life‐history strategies, if some traits do and others fail to respond to altered environments. Altogether, heterogeneity in traits’ plastic and genetic components suggests that life histories are mosaics of trait groups with different capacities for phenotypic and evolutionary change, possibly rendering the formation of ecotypes a slow, incremental, and occasionally nonparallel process.

## AUTHOR CONTRIBUTIONS

AF, TC, and DNR conceived the study. DNR provided the datasets. AF analyzed the data and drafted the manuscript, with input from all authors. All authors contributed to subsequent revisions.

## DATA ARCHIVING

Data associated with this article are available on Dryad (https://doi.org/10.5061/dryad.547d7wm9n; Felmy et al. 2022).

## CONFLICT OF INTEREST

The authors declare no conflict of interest.

Associate Editor: T. Ezard

Handling Editor: A. McAdam

## Supporting information

Supporting Methods 1: Datasets
Supporting Methods 2: Laboratory rearing protocol
Supporting Methods 3: Measurement of life‐history traits
Supporting Methods 4: Statistical analysis of differences in food levels between datasets
Supporting Methods 5: Statistical analysis of phenotypic plasticity and genetic differentiation – datasets analysed jointly
Supporting Methods 6: Statistical analysis of phenotypic plasticity and genetic differentiation – datasets analysed separately
Supporting Results 1: Differences in food levels between datasets
Figure S1. Map of sampling localities in northeastern Trinidad, West Indies.
Figure S2. Wet weight of female guppies in four datasets, illustrating consistent effects of experimental food treatments yet differences in food levels between datasets.
Figure S3. Drainage‐specific plasticity and genetic differentiation in female length at birth 1 (len1).
Figure S4. Drainage‐specific plasticity and genetic differentiation in female length at birth 3 (len3).
Figure S5. Drainage‐specific plasticity and genetic differentiation in male length at sexual maturity (lenmat).
Figure S6. Drainage‐specific plasticity and genetic differentiation in the mean dry weight of new‐born offspring in litter 2 (mnemb2).
Figure S7. Drainage‐specific plasticity and genetic differentiation in female age at first birth (agepart1).
Figure S8. Drainage‐specific plasticity and genetic differentiation in male age at sexual maturity (agemat).
Figure S9. Drainage‐specific plasticity and genetic differentiation in the mean percentage fat of new‐born offspring in litter 2 (mnembfat2).
Figure S10. Phenotypic correlations between traits when computed across treatment combinations.
Table S1. Number of experimental fish per locality and dataset.
Table S2. Analysis of variance with repeated measures of female growth until the birth of litter 2 (wt0‐wt2).
Table S3. Analysis of variance with repeated measures of female growth until the birth of litter 3 (wt0‐wt3).
Table S4. Linear mixed‐effects model on female age at the beginning of the experiment (age0).
Table S5. Linear mixed‐effects model on male age at the beginning of the experiment (age0m).
Table S6. Linear mixed‐effects model on male age at sexual maturity (agemat).
Table S7. Linear mixed‐effects model on female age at first birth (agepart1).
Table S8. Linear mixed‐effects model on female age at second birth (agepart2).
Table S9. Linear mixed‐effects model on female age at third birth (agepart3).
Table S10. Linear mixed‐effects model on the percentage fat in a female's total tissues (fat).
Table S11. Linear mixed‐effects model on the inter‐birth interval 1 (intrvl1).
Table S12. Linear mixed‐effects model on the inter‐birth interval 2 (intrvl2).
Table S13. Linear mixed‐effects model on female standard length at birth 1 (len1).
Table S14. Linear mixed‐effects model on female standard length at birth 2 (len2).
Table S15. Linear mixed‐effects model on female standard length at birth 3 (len3).
Table S16. Linear mixed‐effects model on male standard length at sexual maturity (lenmat).
Table S17. Linear mixed‐effects model on the mean dry weight of new‐born offspring in litter 1 (mnemb1).
Table S18. Linear mixed‐effects model on the mean dry weight of new‐born offspring in litter 2 (mnemb2).
Table S19. Linear mixed‐effects model on the mean dry weight of new‐born offspring in litter 3 (mnemb3).
Table S20. Linear mixed‐effects model on the mean percentage fat in new‐born offspring in litter 1 (mnembfat1).
Table S21. Linear mixed‐effects model on the mean percentage fat in new‐born offspring in litter 2 (mnembfat2).
Table S22. Linear mixed‐effects model on the mean percentage fat in new‐born offspring in litter 3 (mnembfat3).
Table S23. Linear mixed‐effects model on the number of offspring in litter 1 (n1).
Table S24. Linear mixed‐effects model on the maternal‐weight‐adjusted number of offspring in litter 1 (n1_wt1adj).
Table S25. Linear mixed‐effects model on the number of offspring in litter 2 (n2).
Table S26. Linear mixed‐effects model on the maternal‐weight‐adjusted number of offspring in litter 2 (n2_wt2adj).
Table S27. Linear mixed‐effects model on the number of offspring in litter 3 (n3).
Table S28. Linear mixed‐effects model on the maternal‐weight‐adjusted number of offspring in litter 3 (n3_wt3adj).
Table S29. Linear mixed‐effects model on the reproductive allotment (repall).
Table S30. Linear mixed‐effects model on the percentage fat in a female's reproductive tissues (repfat).
Table S31. Linear mixed‐effects model on the dry weight of a female's reproductive tissues (repwt).
Table S32. Linear mixed‐effects model on the percentage fat in a female's somatic tissues (somfat).
Table S33. Linear mixed‐effects model on the dry weight of a female's somatic tissues (somwt).
Table S34. Linear mixed‐effects model on the female wet weight at the beginning of the experiment (wt0).
Table S35. Linear mixed‐effects model on the male wet weight at the beginning of the experiment (wt0m).
Table S36. Linear mixed‐effects model on the female wet weight at birth 1 (wt1).
Table S37. Linear mixed‐effects model on the female wet weight at birth 2 (wt2).
Table S38. Linear mixed‐effects model on the female wet weight at birth 3 (wt3).
Table S39. Linear mixed‐effects model on the male wet weight at sexual maturity (wtmat).
