# Supplementary material for: Life histories as mosaics: Plastic and genetic components differ among traits that underpin life‐history strategies
Source: Evolution. 2022 Feb 6;76(3):585–604. doi: 10.1111/evo.14440 (PMC9303950; doi:10.1111/evo.14440)
Supplement: Supplementary file 1 — Supporting Methods 1: Datasets Supporting Methods 2: Laboratory rearing protocol Supporting Methods 3: Measurement of life‐history traits Supporting Methods 4: Statistical analysis of differences in food levels between datasets Supporting Methods 5: Statistical analysis of phenotypic plasticity and genetic differentiation – datasets analysed jointly Supporting Methods 6: Statistical analysis of phenotypic plasticity and genetic differentiation – datasets analysed separately Supporting Results 1: Differences in food levels between datasets Figure S1. Map of sampling localities in northeastern Trinidad, West Indies. Figure S2. Wet weight of female guppies in four datasets, illustrating consistent effects of experimental food treatments yet differences in food levels between datasets. Figure S3. Drainage‐specific plasticity and genetic differentiation in female length at birth 1 (len1). Figure S4. Drainage‐specific plasticity and genetic differentiation in female length at birth 3 (len3). Figure S5. Drainage‐specific plasticity and genetic differentiation in male length at sexual maturity (lenmat). Figure S6. Drainage‐specific plasticity and genetic differentiation in the mean dry weight of new‐born offspring in litter 2 (mnemb2). Figure S7. Drainage‐specific plasticity and genetic differentiation in female age at first birth (agepart1). Figure S8. Drainage‐specific plasticity and genetic differentiation in male age at sexual maturity (agemat). Figure S9. Drainage‐specific plasticity and genetic differentiation in the mean percentage fat of new‐born offspring in litter 2 (mnembfat2). Figure S10. Phenotypic correlations between traits when computed across treatment combinations. Table S1. Number of experimental fish per locality and dataset. Table S2. Analysis of variance with repeated measures of female growth until the birth of litter 2 (wt0‐wt2). Table S3. Analysis of variance with repeated measures of female growth until the birth of litter 3 (wt0‐wt3). Table S4. Li [file EVO-76-585-s001.docx]

Supporting Information for

**Life histories as mosaics: plastic and genetic components differ among traits that underpin life-history strategies**

Anja Felmy, David N. Reznick, Joseph Travis, Tomos Potter, Tim Coulson

Table of Contents

[Supporting Methods 4](#_Toc91159917)

[Supporting Methods 1: Datasets 4](#_Toc91159918)

[Supporting Methods 2: Laboratory rearing protocol 4](#_Toc91159919)

[Supporting Methods 3: Measurement of life-history traits 6](#_Toc91159920)

[Supporting Methods 4: Statistical analysis of differences in food levels between datasets 7](#_Toc91159921)

[Supporting Methods 5: Statistical analysis of phenotypic plasticity and genetic differentiation – datasets analysed jointly 9](#_Toc91159922)

[Supporting Methods 6: Statistical analysis of phenotypic plasticity and genetic differentiation – datasets analysed separately 11](#_Toc91159923)

[Supporting Results 12](#_Toc91159924)

[Supporting Results 1: Differences in food levels between datasets 12](#_Toc91159925)

[Supporting Figures 14](#_Toc91159926)

[Figure S1. Map of sampling localities in northeastern Trinidad, West Indies. 14](#_Toc91159927)

[Figure S2. Wet weight of female guppies in four datasets, illustrating consistent effects of experimental food treatments yet differences in food levels between datasets. 15](#_Toc91159928)

[Figure S3. Drainage-specific plasticity and genetic differentiation in female length at birth 1 (len1). 16](#_Toc91159929)

[Figure S4. Drainage-specific plasticity and genetic differentiation in female length at birth 3 (len3). 17](#_Toc91159930)

[Figure S5. Drainage-specific plasticity and genetic differentiation in male length at sexual maturity (lenmat). 18](#_Toc91159931)

[Figure S6. Drainage-specific plasticity and genetic differentiation in the mean dry weight of new-born offspring in litter 2 (mnemb2). 19](#_Toc91159932)

[Figure S7. Drainage-specific plasticity and genetic differentiation in female age at first birth (agepart1). 20](#_Toc91159933)

[Figure S8. Drainage-specific plasticity and genetic differentiation in male age at sexual maturity (agemat). 21](#_Toc91159934)

[Figure S9. Drainage-specific plasticity and genetic differentiation in the mean percentage fat of new-born offspring in litter 2 (mnembfat2). 22](#_Toc91159935)

[Figure S10. Phenotypic correlations between traits when computed across treatment combinations. 23](#_Toc91159936)

[Supporting Tables 24](#_Toc91159937)

[Table S1. Number of experimental fish per locality and dataset. 24](#_Toc91159938)

[Table S2. Analysis of variance with repeated measures of female growth until the birth of litter 2 (wt0-wt2). 25](#_Toc91159939)

[Table S3. Analysis of variance with repeated measures of female growth until the birth of litter 3 (wt0-wt3). 28](#_Toc91159940)

[Table S4. Linear mixed-effects model on female age at the beginning of the experiment (age0). 30](#_Toc91159941)

[Table S5. Linear mixed-effects model on male age at the beginning of the experiment (age0m). 31](#_Toc91159942)

[Table S6. Linear mixed-effects model on male age at sexual maturity (agemat). 32](#_Toc91159943)

[Table S7. Linear mixed-effects model on female age at first birth (agepart1). 33](#_Toc91159944)

[Table S8. Linear mixed-effects model on female age at second birth (agepart2). 34](#_Toc91159945)

[Table S9. Linear mixed-effects model on female age at third birth (agepart3). 35](#_Toc91159946)

[Table S10. Linear mixed-effects model on the percentage fat in a female’s total tissues (fat). 36](#_Toc91159947)

[Table S11. Linear mixed-effects model on the inter-birth interval 1 (intrvl1). 37](#_Toc91159948)

[Table S12. Linear mixed-effects model on the inter-birth interval 2 (intrvl2). 38](#_Toc91159949)

[Table S13. Linear mixed-effects model on female standard length at birth 1 (len1). 39](#_Toc91159950)

[Table S14. Linear mixed-effects model on female standard length at birth 2 (len2). 40](#_Toc91159951)

[Table S15. Linear mixed-effects model on female standard length at birth 3 (len3). 41](#_Toc91159952)

[Table S16. Linear mixed-effects model on male standard length at sexual maturity (lenmat). 42](#_Toc91159953)

[Table S17. Linear mixed-effects model on the mean dry weight of new-born offspring in litter 1 (mnemb1). 43](#_Toc91159954)

[Table S18. Linear mixed-effects model on the mean dry weight of new-born offspring in litter 2 (mnemb2). 44](#_Toc91159955)

[Table S19. Linear mixed-effects model on the mean dry weight of new-born offspring in litter 3 (mnemb3). 45](#_Toc91159956)

[Table S20. Linear mixed-effects model on the mean percentage fat in new-born offspring in litter 1 (mnembfat1). 46](#_Toc91159957)

[Table S21. Linear mixed-effects model on the mean percentage fat in new-born offspring in litter 2 (mnembfat2). 47](#_Toc91159958)

[Table S22. Linear mixed-effects model on the mean percentage fat in new-born offspring in litter 3 (mnembfat3). 48](#_Toc91159959)

[Table S23. Linear mixed-effects model on the number of offspring in litter 1 (n1). 49](#_Toc91159960)

[Table S24. Linear mixed-effects model on the maternal-weight-adjusted number of offspring in litter 1 (n1_wt1adj). 50](#_Toc91159961)

[Table S25. Linear mixed-effects model on the number of offspring in litter 2 (n2). 51](#_Toc91159962)

[Table S26. Linear mixed-effects model on the maternal-weight-adjusted number of offspring in litter 2 (n2_wt2adj). 52](#_Toc91159963)

[Table S27. Linear mixed-effects model on the number of offspring in litter 3 (n3). 53](#_Toc91159964)

[Table S28. Linear mixed-effects model on the maternal-weight-adjusted number of offspring in litter 3 (n3_wt3adj). 54](#_Toc91159965)

[Table S29. Linear mixed-effects model on the reproductive allotment (repall). 55](#_Toc91159966)

[Table S30. Linear mixed-effects model on the percentage fat in a female’s reproductive tissues (repfat). 56](#_Toc91159967)

[Table S31. Linear mixed-effects model on the dry weight of a female’s reproductive tissues (repwt). 57](#_Toc91159968)

[Table S32. Linear mixed-effects model on the percentage fat in a female’s somatic tissues (somfat). 58](#_Toc91159969)

[Table S33. Linear mixed-effects model on the dry weight of a female’s somatic tissues (somwt). 59](#_Toc91159970)

[Table S34. Linear mixed-effects model on the female wet weight at the beginning of the experiment (wt0). 60](#_Toc91159971)

[Table S35. Linear mixed-effects model on the male wet weight at the beginning of the experiment (wt0m). 61](#_Toc91159972)

[Table S36. Linear mixed-effects model on the female wet weight at birth 1 (wt1). 62](#_Toc91159973)

[Table S37. Linear mixed-effects model on the female wet weight at birth 2 (wt2). 63](#_Toc91159974)

[Table S38. Linear mixed-effects model on the female wet weight at birth 3 (wt3). 64](#_Toc91159975)

[Table S39. Linear mixed-effects model on the male wet weight at sexual maturity (wtmat). 65](#_Toc91159976)

[Supporting References 66](#_Toc91159977)

# Supporting Methods

## Supporting Methods 1: Datasets

We here used four datasets of guppies from both ecotypes that were subjected to a low- and a high-quantity food regime in the laboratory. Three datasets have previously been published fully or in part, while one is published here for the first time. All datasets include fish from multiple sampling localities. The localities, years of collection of wild-caught females, and sample sizes are listed in Table S1 and their locations illustrated in Fig. S1. In combination, the datasets include 708 females and 470 males. Dataset 1 uses fish from an introduction experiment where, in 1981, fast-reproducing guppies from a high-predation locality (“Control”) were transplanted to a low-predation locality (“Introduction”) situated within the same drainage, and both localities were sampled four years post-introduction (for details see Reznick and Bryga, 1987). Dataset 2 includes paired localities of fast- and slow-reproducing guppies from the Madamas, Marianne, and Yarra Rivers. Four of these localities formed part of a larger survey of parallel evolution in the life histories of guppies inhabiting streams with different predation regimes (for details see Reznick and Bryga, 1996). Dataset 3 is unpublished. It contains three fast-reproducing and three slow-reproducing populations from four drainages – the Curaguate River (fast), the Marianne River (fast and slow), the Paria River (two slow), and the Yarra River (fast). Finally, dataset 4 consists of paired localities of fast- and slow-reproducing guppies from the Oropuche and Yarra Rivers, and was created to study female senescence (for details see Reznick et al., 2004, Reznick et al., 2006). While datasets 1 to 3 contain both sexes, dataset 4 is female-only.

## Supporting Methods 2: Laboratory rearing protocol

The laboratory methods used have been described in detail elsewhere (see, e.g., Reznick and Bryga, 1987). Briefly, in all datasets, experimental individuals consisted of the second generation of laboratory-born offspring derived from wild-caught, gravid females. Experimental individuals were kept in maternal sib-groups. Typically, all the offspring from a given mother were reared together in two groups of five in two-gallon tanks that were next to each other on the shelf. When offspring were 26.2 ± 3.6 days old (mean ± SD, min: 19.0, max: 40.0), they were measured, weighed, and sexed. Subsequently, two males and two females in each maternal sib-group were selected for the quantified feeding treatment, and one individual of each sex randomly assigned to a high or low food level, respectively.

The exact food levels used are unknown but differed between datasets. In dataset 1, the low and high food levels were “systematically lower” (Reznick and Bryga, 1987, p. 1374) than in a previous study, in which food availability had been set at a level promoting 50 to 85% of the growth rate observed with *ad libitum* feeding (Reznick, 1983). In dataset 2, the high and low food levels were chosen to sustain 65-70% and 45-50% of the maximum growth rate, respectively (Reznick and Bryga, 1996). These levels had been established in a previous series of experiments, which also showed that uncontrolled food sources (e.g., algae) contribute minimally to growth (Reznick, 1980). No information is available about the food levels used in the unpublished dataset 3. In dataset 4, the food levels were the same as in dataset 2. All fish were fed liver paste in the morning, and a paste made of living brine shrimp nauplii (*Artemia* sp.) in the afternoon, with quantities being controlled volumetrically, to the nearest microlitre, using Hamilton micropipettes. In both food treatments, quantities of food were increased biweekly to accommodate growth.

The four tanks of each maternal sib-group, or two tanks in the case of dataset 4, which does not contain males, were kept together throughout the experiment. They were distributed around the laboratory in a stratified randomised block array to avoid confounding the laboratory microenvironment with sampling locality and experimental food levels. However, as a consequence, maternal identity is confounded with other factors, such as variation across the laboratory in temperature.

## Supporting Methods 3: Measurement of life-history traits

Although some of the traits listed in the following may not usually be considered life-history traits (e.g., percentage fat), we here use the term in a wider sense to include both life-history and life-history-associated traits. For males, the measured phenotypic traits were age, wet weight, and standard length at sexual maturity. For females, we measured the age, wet weight, and standard length when they gave birth to their first, second, and third litter, the first and second inter-birth intervals (i.e., the time between litters 1 and 2, and between litters 2 and 3, respectively), the dry weight of and percentage fat in a female’s somatic and reproductive tissues, the percentage fat in her total tissues, the reproductive allotment, the number of offspring in litters 1, 2 and 3, and the mean dry weight of and mean percentage fat in new-born offspring in litters 1, 2 and 3. As female size contributes to fecundity, we also analysed litter sizes when fitting the postpartum maternal wet weight as a covariate (following Reznick and Bryga, 1987); these maternal-weight-adjusted litter sizes are included as separate traits. In addition, we included the male and female age and wet weight at the beginning of the experiment, when the controlled feeding regime had not yet started, as negative controls for the effect of food.

We consequently collected data on 36 dependent variables. Only dataset 2 includes measurements of all variables; the numbers of variables available from datasets 1, 3 and 4 are 27, 24, and 25, respectively. Dataset 1 lacks all the variables pertaining to a female’s third litter. Consequently, dataset 1 also differs from the other datasets inasmuch as the dry weights and percentage fat of females were measured after they produced their second (not third) litter, and the reproductive allotment calculated based on the second (not third) litter. In dataset 3, all post-mortem measurements of both females and offspring are lacking. Dataset 4 lacks the post-mortem measurements of females, and no data were collected for males.

Male maturity was characterised by the development of the intromittent organ, the gonopodium (metamorphosed anal fin), which was initially checked weekly, then daily as males approached maturity, until the attainment of sexual maturity. Females were mated once a week until they reproduced for the first time, and then again within 24 hours after they gave birth to their first, second and third litters. The offspring in litters one to three were counted and preserved within 12 hours of birth. Females were preserved immediately after bearing their third litter, except in dataset 1, where females were preserved after two litters. *Post mortem*, females were dissected, the gut and gut contents discarded, and the somatic and reproductive tissues oven-dried overnight at 60°C. The tissues were then weighed separately to the nearest 0.1 mg (dry weights). After a series of ether extractions to remove fat deposits, tissues were weighed again (fat-free dry weights). We calculated the percentage fat in a female’s total, somatic and reproductive tissues as the proportion of the dry weight that was lost after the ether extractions, i.e., as the difference between the dry and fat-free dry weights, divided by the dry weights. In the same way, we measured the mean dry weights of and mean percentage fat in offspring in litters 1 to 3. A female’s reproductive allotment was computed by dividing the total dry weight of offspring in her last litter by the sum of her total dry weight and the litter’s total dry weight.

## Supporting Methods 4: Statistical analysis of differences in food levels between datasets

We used analysis of variance with repeated measures to test for differences in female growth rates as a function of the experimental food level, the dataset, the ecotype, and the drainage. The primary goal was to compare the chosen food levels among datasets by using growth as a proxy for the size of food rations. We focused on females because dataset 4 did not include males, and because females were weighed four times and males only twice.

Two analyses were performed, each on a different subset of the data. In the first, which included all four datasets, the dependent variable was the female wet weight at the beginning of the experiment and at the birth of litters 1 and 2. The second analysis additionally included the female wet weight at the birth of litter 3. This analysis only included datasets 2 to 4, as in dataset 1 females were euthanised after producing two litters and hence no data exist on their weight at the birth of litter 3.

We analysed female weights with a repeated measures analysis of variance, using function “aov” in R (R Core Team, 2020). The predictors were food level, age category, dataset, all their interactions including the three-way-interaction, ecotype, the interaction between food level and ecotype, drainage, and maternal identity nested within drainage. Our formula contained individual identity as the single error term, to account for repeated measurements of females. The error term specified two different error strata, with appropriate models fitted within each stratum. We tested the effects of age and all its interactions within individuals, while we tested all the other effects between individuals. We compared models using raw, base-*e* log-transformed and square-root-transformed wet weights. Although the models gave very similar results, we found that a log-transformation resulted in the best fit, as judged from diagnostic plots.

After fitting the two full models, we computed pairwise comparisons to find out where the differences between datasets lay. Due to the strong interactions between datasets, food levels and age categories, each pairwise comparison of datasets was made within a given food level and age category, while using the full model residuals as the error term. This resulted in 36 pairwise comparisons in the first analysis (4 datasets, 3 age categories, 2 food levels), and 24 in the second analysis (3 datasets, 4 age categories, 2 food levels). In analyses of variance, the order in which predictors are fitted is important. To further investigate differences between ecotypes, we therefore ran additional analyses where ecotype was fitted as the first predictor.

## Supporting Methods 5: Statistical analysis of phenotypic plasticity and genetic differentiation – datasets analysed jointly

To find appropriate models, we fitted three models per trait: one each where trait values were raw, base-*e* log-transformed, and square-root-transformed. To avoid undefined values for values of zero of six traits (dry weight of a female’s reproductive tissues, percentage fat in a female’s total and somatic tissues, mean percentage fat in new-born offspring in litters 1 to 3) when performing log transformations, 0.01 was added to all values of these traits before log-transforming them. For the number of offspring in litters 1, 2 and 3, we additionally fitted a generalised linear mixed model with Poisson errors. For traits that are proportions (e.g., percentage fat, reproductive allotment), we additionally fitted a GLMM with binomial errors. We then selected the best-fitting model based on diagnostic plots and model convergence. Only models that converged were selected.

All models included the experimental food treatment (high vs. low), the ecotype (fast- vs. slow-reproducing), and the dataset (1 to 4) as categorical fixed effects. The reference level was high food, the fast-reproducing ecotype, using dataset 2, as only dataset 2 contained all traits. No interactions between fixed effects were fitted in models shown in the main text, but we additionally fitted models including all two-way interactions between food levels, ecotypes, and datasets. In models with interactions, we used the same data transformation (un-, log-, or square-root-transformed) that proved best in models without interactions. For three traits (litter sizes 1 and 2, mean dry weight of embryos in litter 2), the resulting models were singular or non-converging, and so a different data transformation was used (square-root, untransformed, square root, respectively) that allowed the model to converge.

For some traits, our models included covariates. We fitted additional analyses of litter sizes where the postpartum maternal wet weight was used as a covariate and show these maternal-weight-adjusted analyses alongside analyses of raw litter sizes. We also evaluated the male wet weight at the beginning of experiments as a potential covariate for male standard length, wet weight, and age at sexual maturity (following Reznick and Bryga, 1987). Including the covariate did not change the model results, and so weight-unadjusted analyses of these traits are shown. Before fitting models including a covariate, covariates were mean-centred by subtracting the covariate’s average from each individual value of the covariate. This was necessary to obtain intercepts comparable to those of models not containing covariates. Comparable intercepts were required for computing mean-standardised effect sizes.

As random effects we included maternal identity nested within drainage, provided that drainage had five levels or more. For eight traits we had data from four drainages only, and so drainage was fitted as a categorical fixed effect, with maternal identity as the sole random effect. Fitting maternal identity within locality was precluded by the strong collinearity between locality and ecotype. For two traits (male and female age at the beginning of the experiment), maternal identity could not be included as a random effect, as fish from the same mother came from a single litter, and thus were of the same age. Unlike the other datasets, the so-far unpublished dataset 3 contains four localities that are not paired fast- and slow-reproducing populations from a single drainage. The resulting imbalance of the data could potentially affect our results. We therefore repeated our analyses after excluding the unpaired localities for a subset of the traits and found that the results were not substantially different. Consequently, we kept using the full dataset.

Outlier screening revealed three traits (age at the birth of litter 3, inter-birth intervals 1 and 2) that showed moderately influential data points (maximum Cook’s distance: 0.20), and additional models were fitted after excluding these outliers. The results of the outlier-free and original models were very similar, so outliers were retained in our final models.

## Supporting Methods 6: Statistical analysis of phenotypic plasticity and genetic differentiation – datasets analysed separately

We used the same type of data transformation that resulted in the best fit when analysing datasets jointly. Models included the experimental food treatment and the ecotype as fixed effects, and maternal identity as a random effect, except for the age at the beginning of experiments, where maternal identity could not be included as all fish from a given mother were born on the same day. For datasets 2 and 4, containing 3 and 2 drainages, respectively, the drainage was included as a third fixed effect. For dataset 1, containing a single drainage, drainage was not included as a predictor. Dataset 3 contained four drainages, yet we could not include drainage as a predictor because its effects were confounded with differences among ecotypes, with only one of the four drainages being represented by both the fast- and the slow-reproducing ecotype.

# Supporting Results

## Supporting Results 1: Differences in food levels between datasets

In all datasets, females gained weight more slowly under low-food conditions (main effect of food level: *F*_1_ = 301.7, *p* < 0.00001; age x food interaction: *F*_2_ = 102.7, *p* < 0.00001; Tables S2 and S3). However, datasets differed from one another in terms of the mean weight of females (largest difference between datasets computed from raw data: 42% lower weight in dataset 1 vs. 3; main effect of dataset: *F*_3_ = 188.1, *p* < 0.00001), the magnitude to which growth was reduced at the lower food level (range: 16.1% in dataset 3 vs. 33.3% in dataset 4; food x dataset interaction: *F*_3_ = 9.13, *p* < 0.00001), and the shape of growth curves themselves (age x dataset interaction: *F*_6_ = 137.5, *p* < 0.00001). When including all four ages at which females were weighed in datasets 2-4, datasets also differed in the extent to which the food levels resulted in progressively larger weight differences as fish aged (three-way interaction between food, age, and dataset: *F*_6_ = 7.92, *p* < 0.00001).

Pairwise comparisons of datasets offered more insight into the effects of variation in food levels (Tables S2 and S3). Dataset 1 had the highest food levels at the start of experiments, but these levels increased very slowly, resulting in fish receiving substantially less food in this compared to other datasets. Dataset 2 had the second-lowest food levels after the start of experiments, followed by dataset 4, which additionally had a particularly pronounced contrast between the high and low food levels as females grew older. In the unpublished dataset 3, food levels were highest by far.

These results were corrected for the effects of drainage (all datasets: *F*_5_ = 34.6, *p* < 0.00001; datasets 2-4: *F*_5_ = 29.7, *p* < 0.00001), maternal identity nested within drainage (all datasets: *F*_343_ = 1.8, *p* < 0.00001; datasets 2-4: *F*_298_ = 1.7, *p* = 0.00001), and ecotype (all datasets: *F*_1_ = 12.7, *p* = 0.00043; datasets 2-4: *F*_1_ = 19.4, *p* = 0.00002, Tables S2 and S3). While the former two effects were significant, differences between ecotypes were only significant when we included all four weights per female, thus excluding dataset 1. These results were very similar when fitting ecotype as the first predictor in either model (all dataset: *F*_1_ = 8.3, *p* = 0.00413; datasets 2-4: *F*_1_ = 15.8, *p* = 0.00009). Across all four datasets, females from the slow-reproducing ecotype were 5.9% heavier than those from the fast-reproducing ecotype.

# Supporting Figures

| 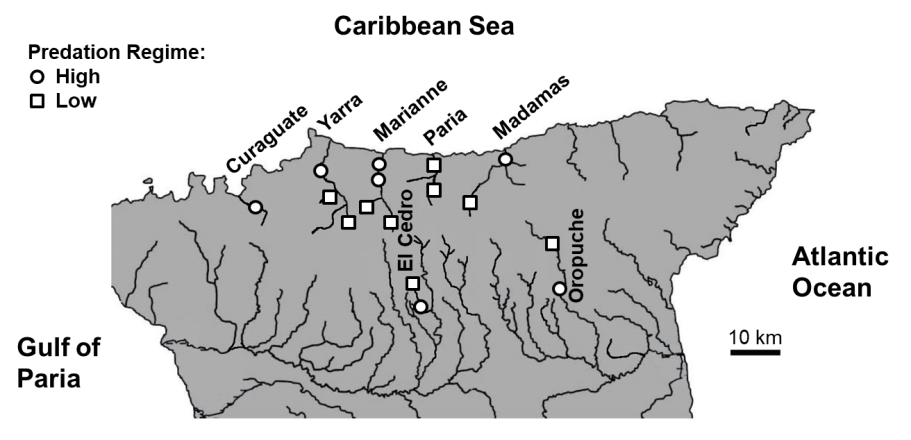 | 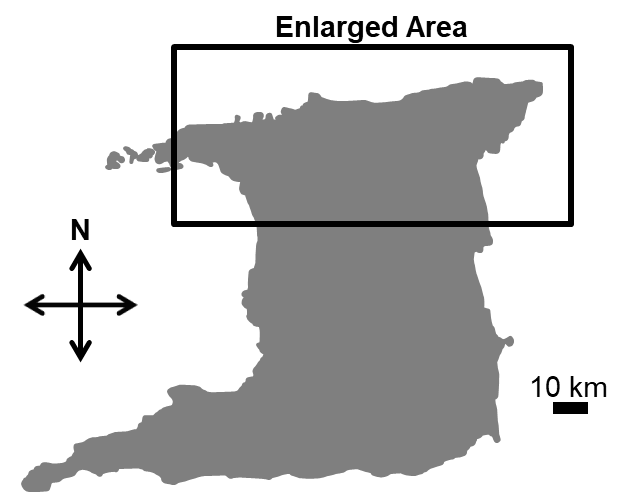 |
| --- | --- |

## **Figure S1**. Map of sampling localities in northeastern Trinidad, West Indies.

High-predation localities are inhabited by the fast- and low-predation localities by the slow-reproducing ecotype. Modified from Reznick and Travis (2019).

| 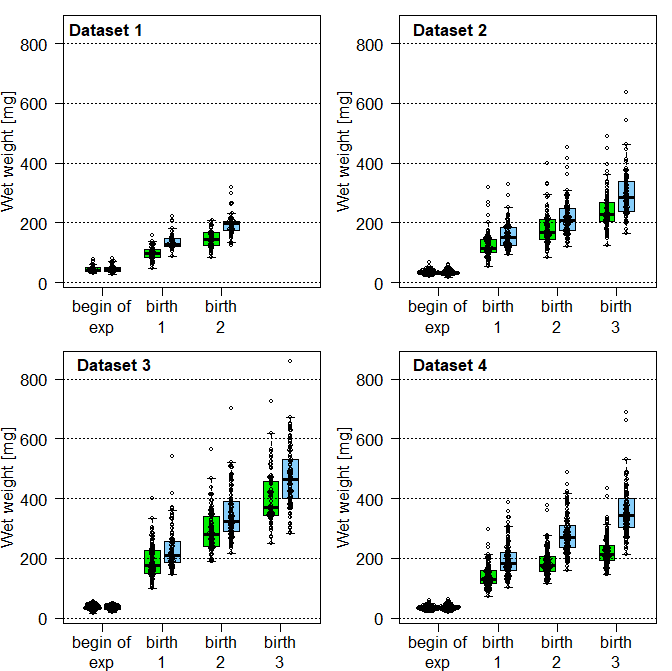 |
| --- |

## **Figure S2. Wet weight of female guppies in four datasets, illustrating consistent effects of experimental food treatments yet differences in food levels between datasets.**

Females were either subjected to a low-quantity diet (in green) or to a high-quantity diet (in blue) and were weighed at the beginning of the controlled food treatment (mean ± SD age: 26.8 ± 3.8 days), and when they gave birth for the first, second, and third time. Up to four data points may therefore stem from the same individual. For model results, see Tables S2 and S3.

| 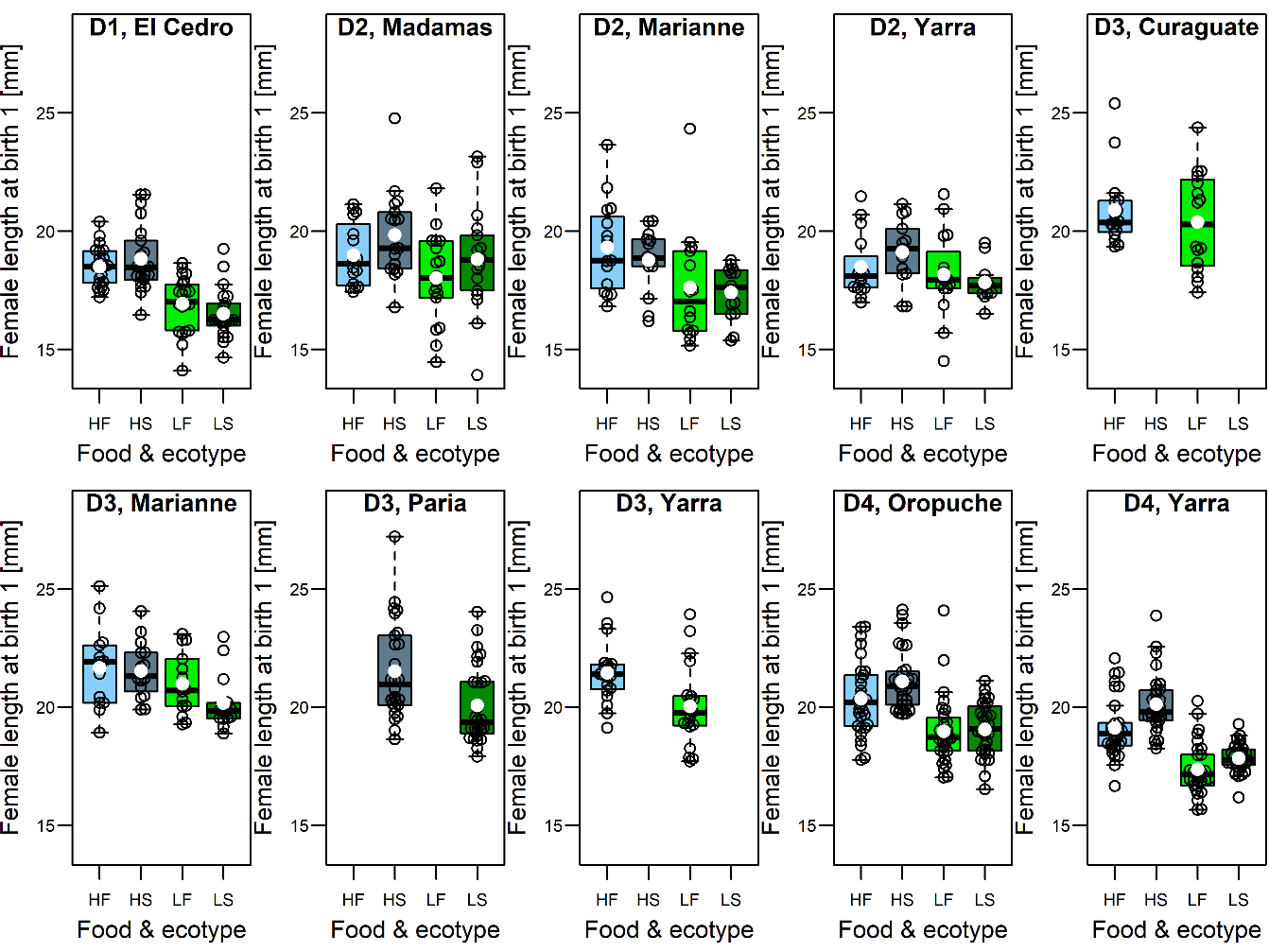 |
| --- |

## Figure S3. Drainage-specific plasticity and genetic differentiation in female length at birth 1 (len1).

In the combined dataset, this trait showed significant plasticity with respect to food levels yet no genetic differentiation between ecotypes. However, there was some variation to these patterns within drainages. White circles denote mean values of categorical predictor levels. D1-D4: dataset 1-4; HF: high food, fast-reproducing ecotype; HS: high food, slow-reproducing ecotype; LF: low food, fast-reproducing ecotype; LS: slow-reproducing ecotype.

| 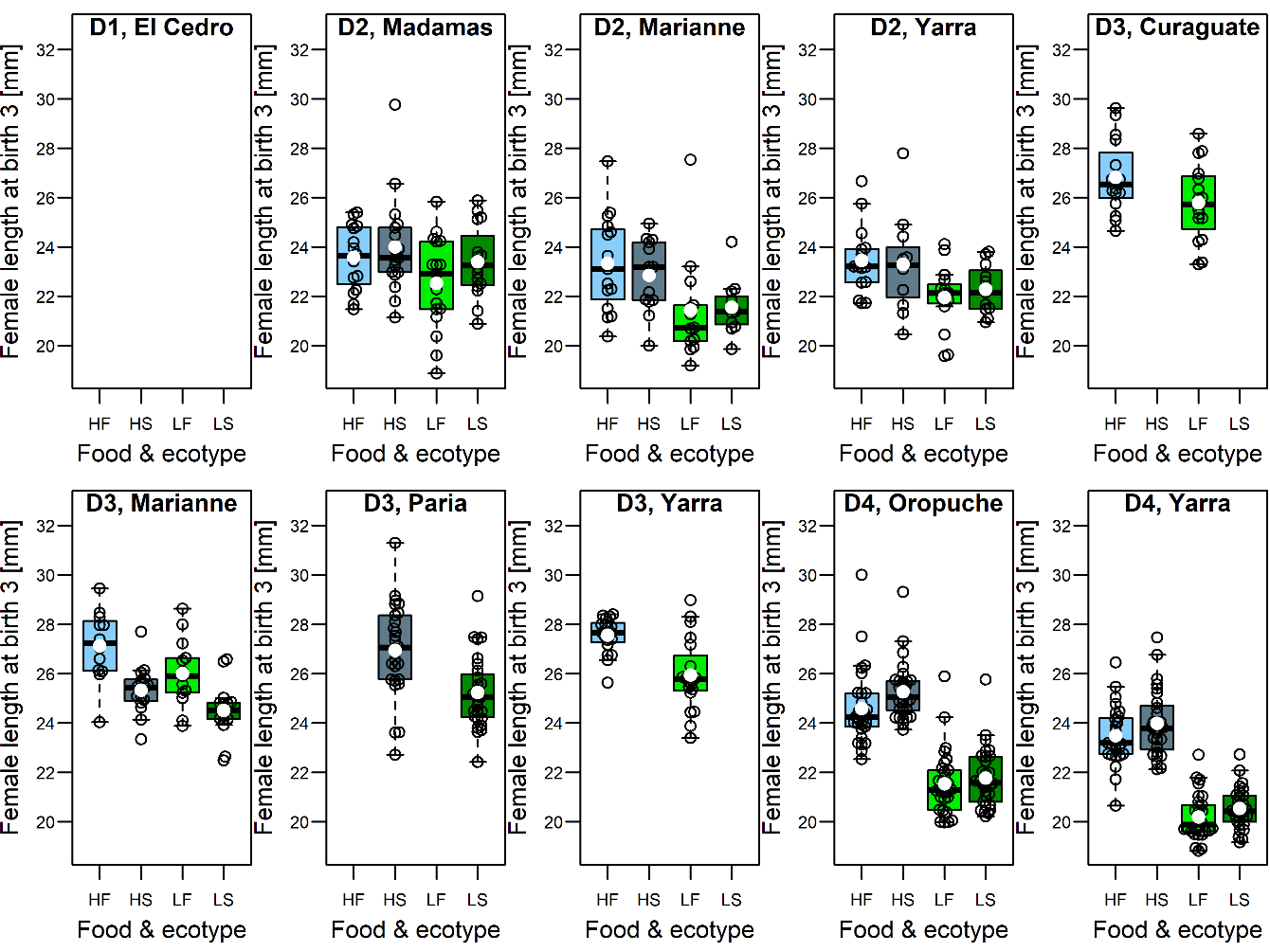 |
| --- |

## Figure S4. Drainage-specific plasticity and genetic differentiation in female length at birth 3 (len3).

In the combined dataset, this trait showed significant plasticity with respect to food levels yet no genetic differentiation between ecotypes. However, there was some variation to these patterns within drainages. White circles denote mean values of categorical predictor levels. The trait was not measured in dataset 1. D1-D4: dataset 1-4; HF: high food, fast-reproducing ecotype; HS: high food, slow-reproducing ecotype; LF: low food, fast-reproducing ecotype; LS: slow-reproducing ecotype.

| 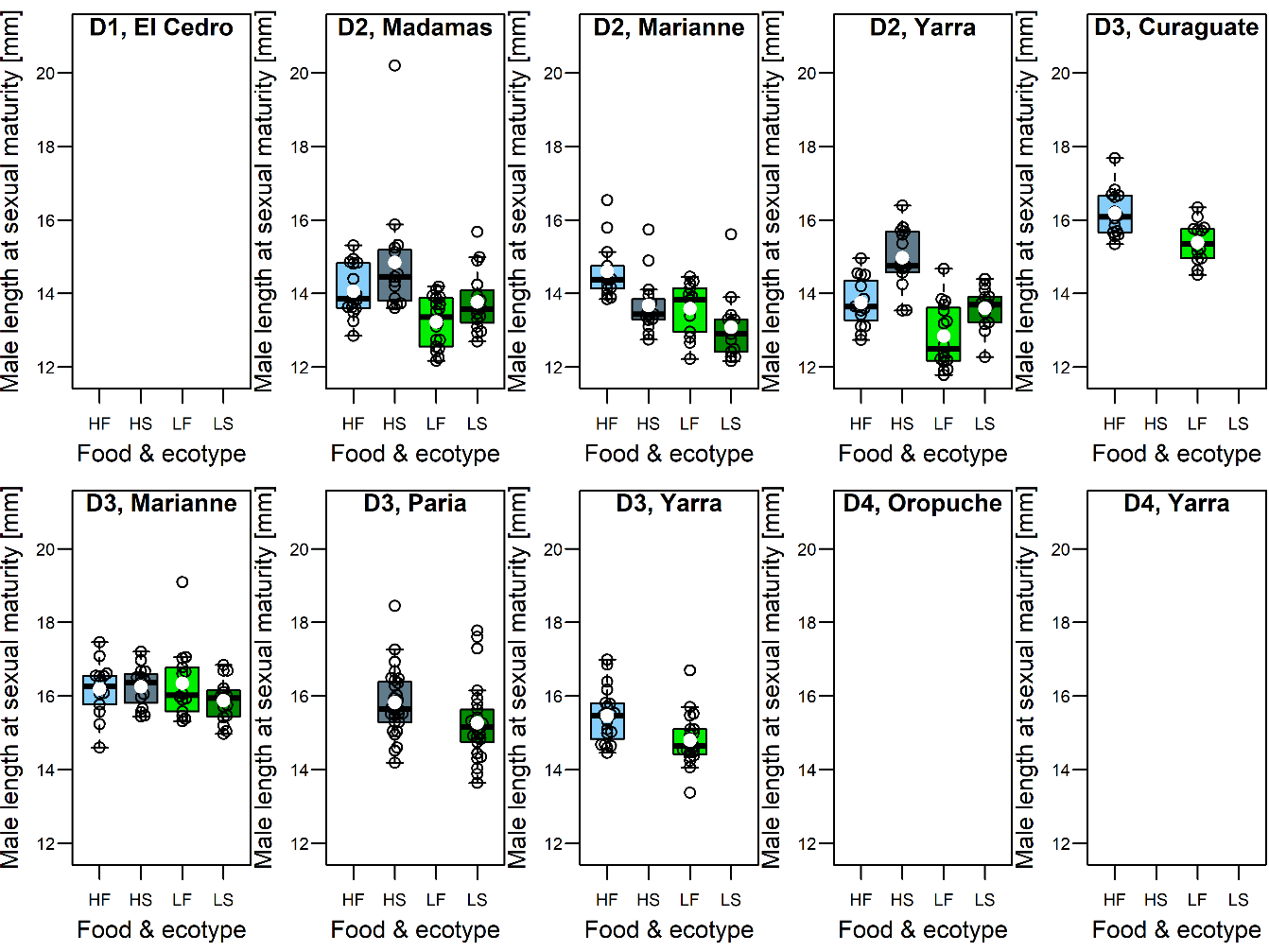 |
| --- |

## Figure S5. Drainage-specific plasticity and genetic differentiation in male length at sexual maturity (lenmat).

In the combined dataset, this trait showed significant plasticity with respect to food levels yet no genetic differentiation between ecotypes. However, there was some variation to these patterns within drainages. White circles denote mean values of categorical predictor levels. The trait was not measured in datasets 1 and 4. D1-D4: dataset 1-4; HF: high food, fast-reproducing ecotype; HS: high food, slow-reproducing ecotype; LF: low food, fast-reproducing ecotype; LS: slow-reproducing ecotype.

| 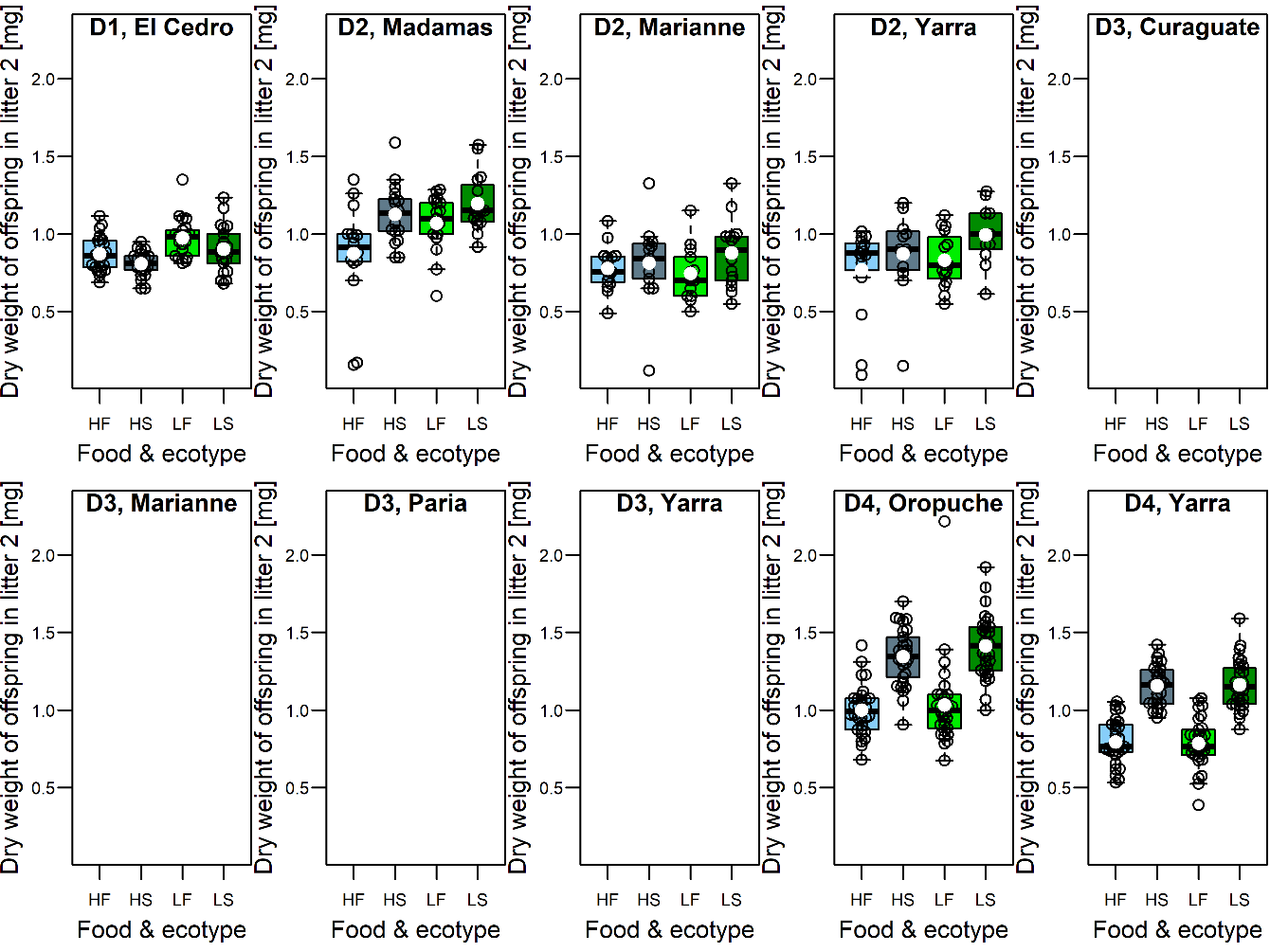 |
| --- |

## Figure S6. Drainage-specific plasticity and genetic differentiation in the mean dry weight of new-born offspring in litter 2 (mnemb2).

In the combined dataset, this trait showed significant differentiation between ecotypes yet no plasticity with respect to food levels. However, there was some variation to these patterns within drainages. White circles denote mean values of categorical predictor levels. The trait was not measured in dataset 3. D1-D4: dataset 1-4; HF: high food, fast-reproducing ecotype; HS: high food, slow-reproducing ecotype; LF: low food, fast-reproducing ecotype; LS: slow-reproducing ecotype.

| 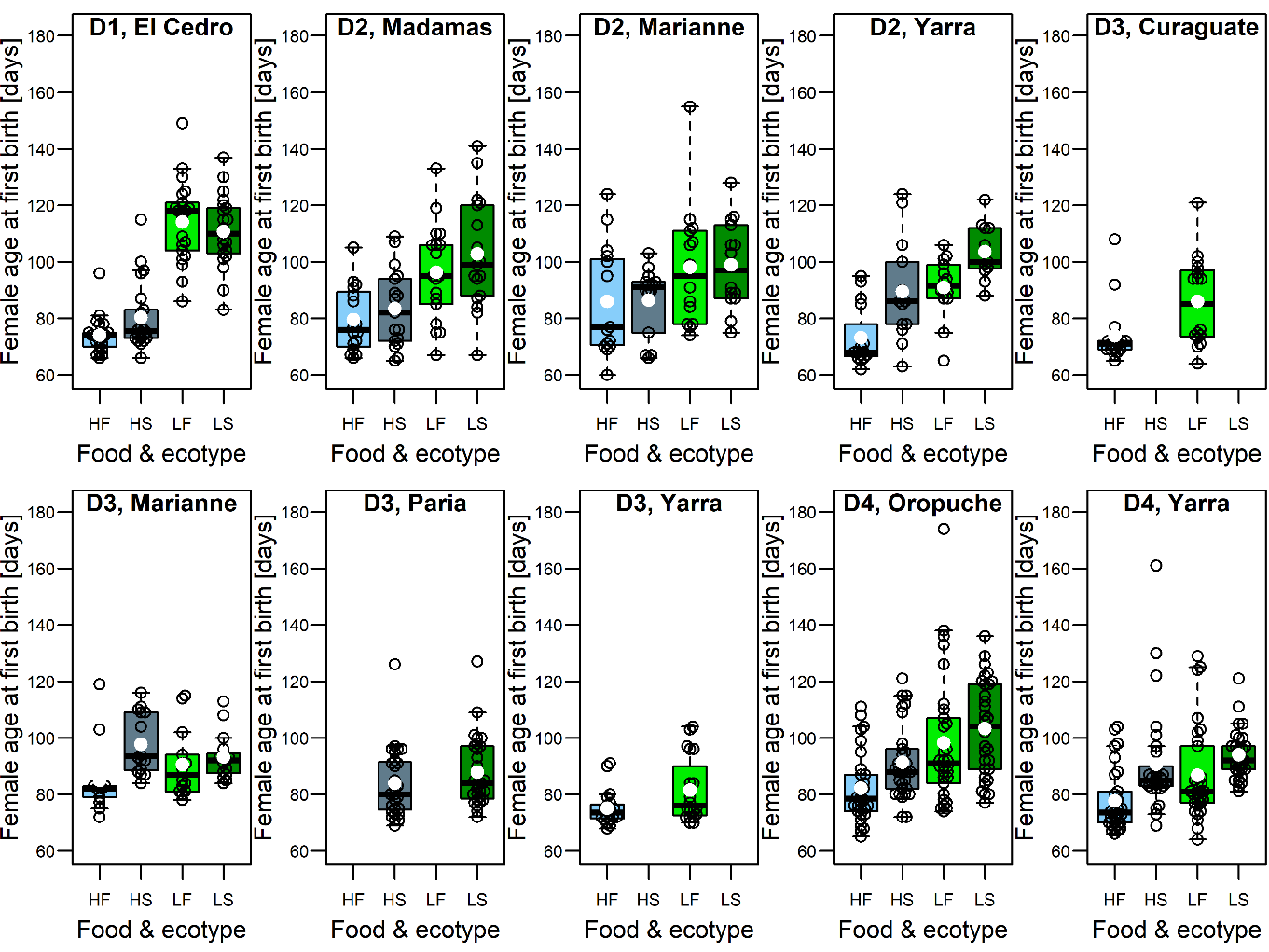 |
| --- |

## Figure S7. Drainage-specific plasticity and genetic differentiation in female age at first birth (agepart1).

In the combined dataset, this trait showed significant plasticity with respect to food levels and significant genetic differentiation between ecotypes. However, there was some variation to these patterns within drainages. White circles denote mean values of categorical predictor levels. D1-D4: dataset 1-4; HF: high food, fast-reproducing ecotype; HS: high food, slow-reproducing ecotype; LF: low food, fast-reproducing ecotype; LS: slow-reproducing ecotype.

| 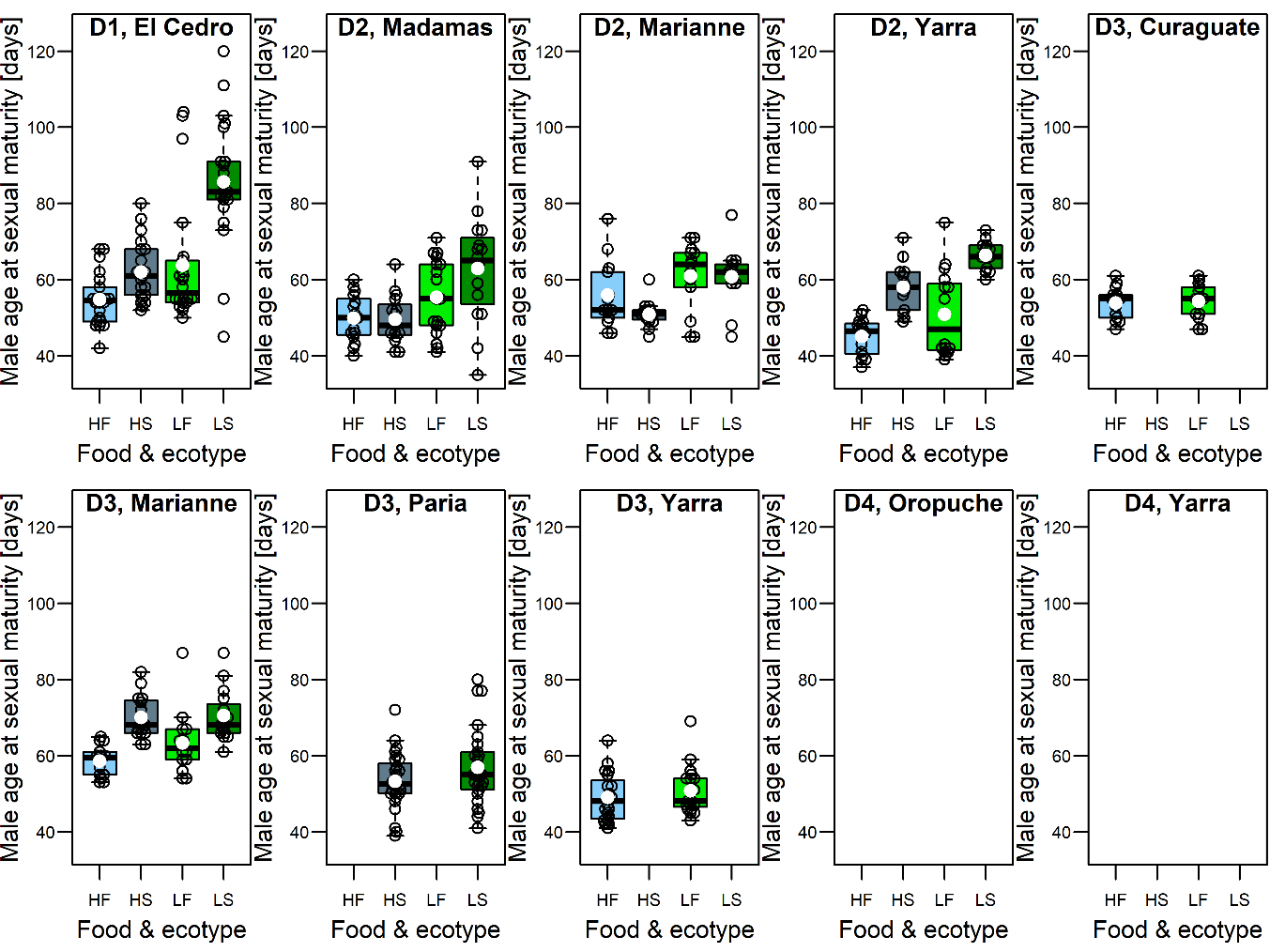 |
| --- |

## Figure S8. Drainage-specific plasticity and genetic differentiation in male age at sexual maturity (agemat).

In the combined dataset, this trait showed significant plasticity with respect to food levels and significant genetic differentiation between ecotypes. However, there was some variation to these patterns within drainages. White circles denote mean values of categorical predictor levels. The trait was not measured in dataset 4. D1-D4: dataset 1-4; HF: high food, fast-reproducing ecotype; HS: high food, slow-reproducing ecotype; LF: low food, fast-reproducing ecotype; LS: slow-reproducing ecotype.

| 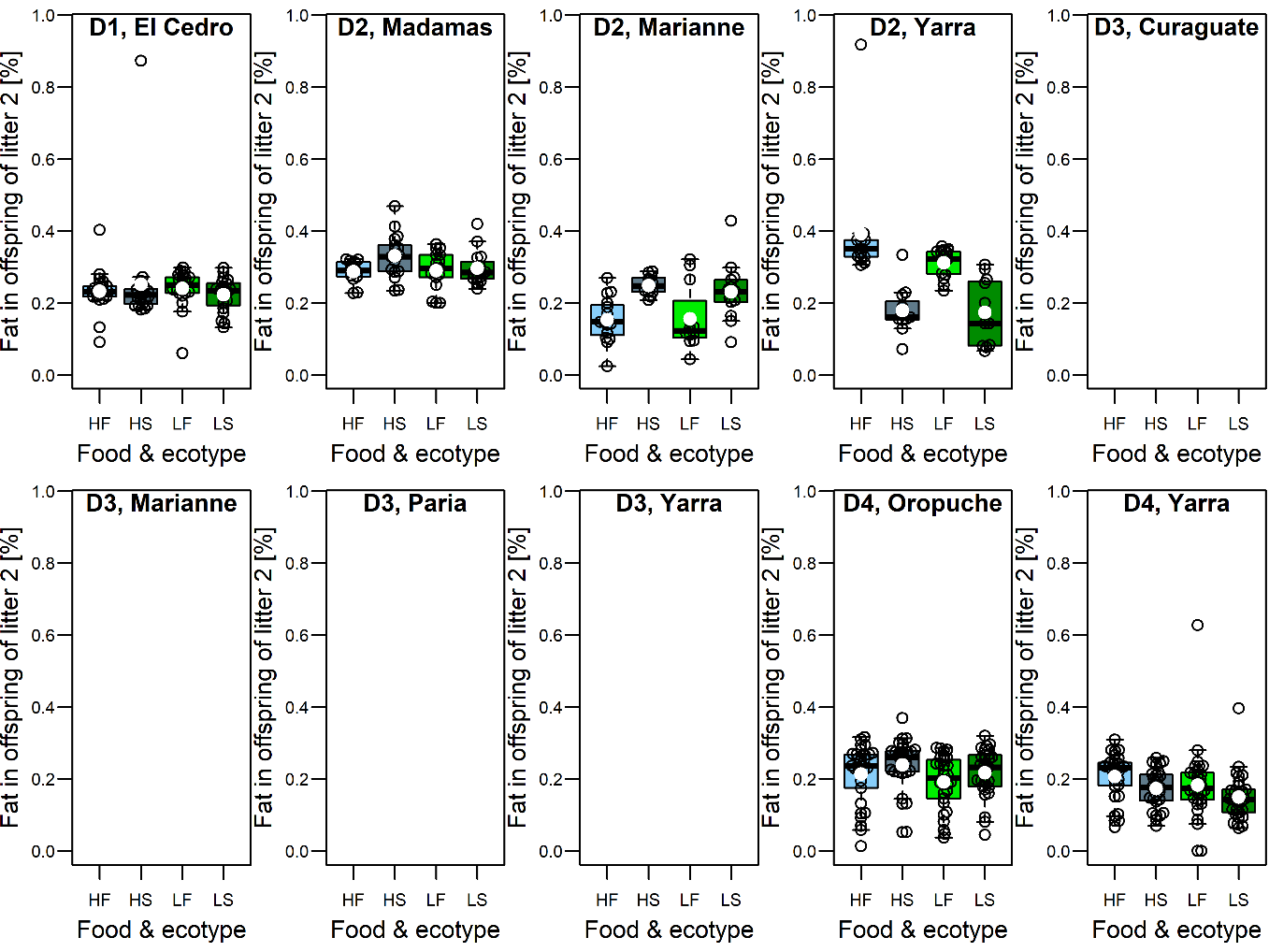 |
| --- |

## Figure S9. Drainage-specific plasticity and genetic differentiation in the mean percentage fat of new-born offspring in litter 2 (mnembfat2).

In the combined dataset, this trait showed neither significant plasticity with respect to food levels nor significant genetic differentiation between ecotypes. However, there was some variation to these patterns within drainages. White circles denote mean values of categorical predictor levels. The trait was not measured in dataset 3. D1-D4: dataset 1-4; HF: high food, fast-reproducing ecotype; HS: high food, slow-reproducing ecotype; LF: low food, fast-reproducing ecotype; LS: slow-reproducing ecotype.

| 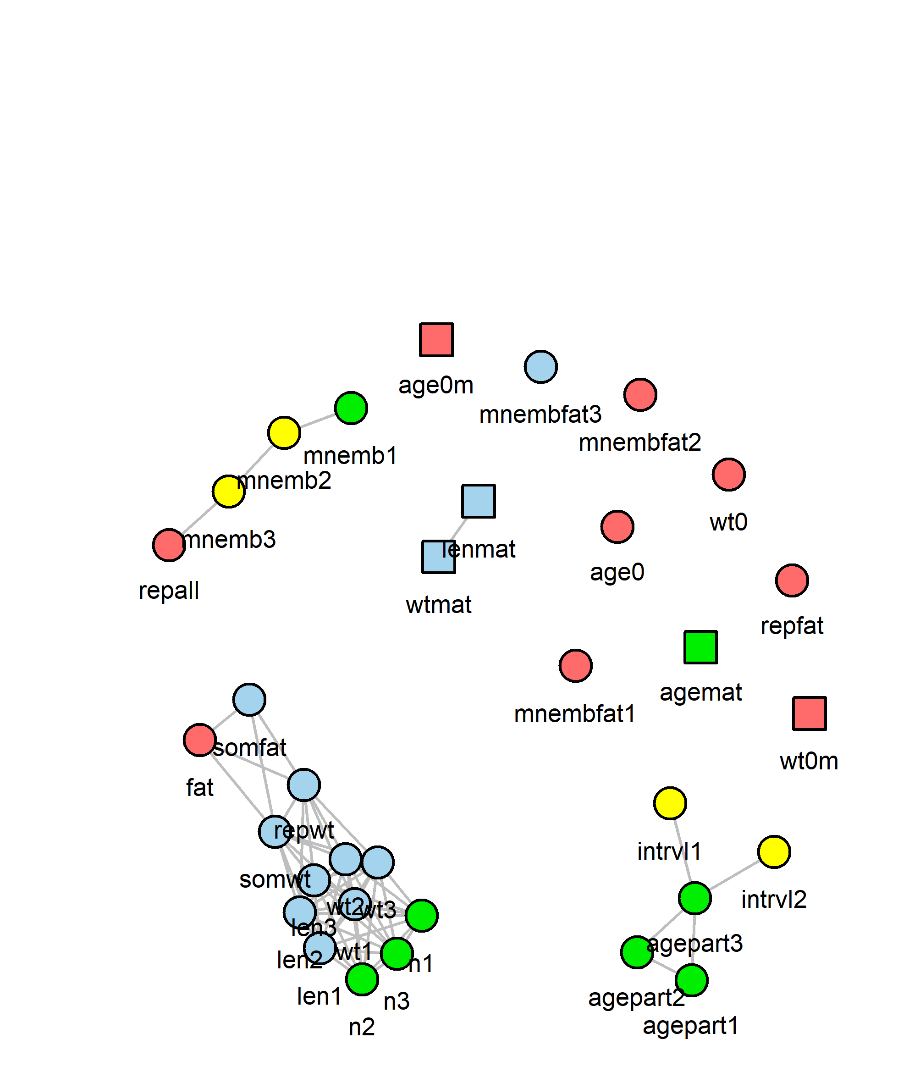 |
| --- |

## Figure S10. Phenotypic correlations between traits when computed across treatment combinations.

Correlations were calculated across all individuals, irrespective of ecotype and food level. The colouring indicates whether traits had significant plastic and genetic components (green), only plastic components (blue), only genetic components (yellow), or neither (red). Grey lines connect traits with pairwise Pearson product-moment correlation coefficients of *r* ≥ 0.5. The correlation between traits measured in females (circles) and traits measured in males (squares) is *r* = 0 by definition. The shorter the connecting line between two traits, the stronger is their correlation. The position of traits and trait clusters relative to one another is irrelevant. Trait abbreviations are explained in Table 3.

# Supporting Tables

## Table S1. Number of experimental fish per locality and dataset.

| Drainage | Locality | Ecotype | Dataset | Year | Experimental fish | |
| --- | --- | --- | --- | --- | --- | --- |
|  |  |  |  |  | Females | Males |
| Curaguate (N) | Curaguate | Fast | 3 | 1996 | 32 | 32 |
| El Cedro (S) | Control | Fast | 1 | 1985 | 44 | 44 |
|  | Introduction | Slow | 1 | 1985 | 44 | 42 |
| Madamas (N) | Madamas | Fast | 2 | 1991 | 36 | 36 |
|  | Tapana-Aqui (tributary) | Slow | 2 | 1991 | 34 | 34 |
| Marianne (N) | Marianne 1 | Fast | 2 | 1990 | 30 | 30 |
|  | Marianne 2 | Fast | 3 | 1996 | 27 | 28 |
|  | Marianne (tributary) | Slow | 2 | 1990 | 31 | 32 |
|  | Marianito (tributary) | Slow | 3 | 1996 | 32 | 32 |
| Oropuche (S) | Oropuche | Fast | 4 | 1998 | 60 | 0 |
|  | Campo (tributary) | Slow | 4 | 1998 | 60 | 0 |
| Paria (N) | Paria | Slow | 3 | 1996 | 30 | 30 |
|  | Paria (tributary) | Slow | 3 | 1996 | 28 | 28 |
| Yarra (N) | Yarra | Fast | 2 | 1990 | 32 | 32 |
|  |  | Fast | 3 | 1996 | 40 | 40 |
|  |  | Fast | 4 | 1998 | 60 | 0 |
|  | Limon 1 (tributary) | Slow | 2 | 1990 | 30 | 30 |
|  | Limon 2 (tributary) | Slow | 4 | 1998 | 58 | 0 |

The year of collection of field-caught ancestors is provided in brackets. Experimental fish are second-generation laboratory-reared offspring of wild-caught fish. Ecotype: pace of reproduction, where a fast pace is linked to high-predation habitats and a slow pace to low-predation habitats; N and S: north and south slope of the Northern Range Mountains of Trinidad; f: number of female experimental fish; m: number of male experimental fish.

## **Table S2.** Analysis of variance with repeated measures of female growth until the birth of litter 2 (wt0-wt2).

|  | Df | | Sum Sq | Mean Sq | *F*-value | *p*-value |  |
| --- | --- | --- | --- | --- | --- | --- | --- |
| Error: between individuals | | |  |  |  |  |  |
| Food | 1 | | 17.64 | 17.64 | 301.66 | < 0.00001 |  |
| Dataset | 3 | | 32.98 | 10.99 | 188.06 | < 0.00001 |  |
| Ecotype | 1 | | 0.74 | 0.74 | 12.69 | 0.00043 |  |
| Drainage | 5 | | 10.11 | 2.02 | 34.60 | < 0.00001 |  |
| Food x Dataset | 3 | | 1.60 | 0.53 | 9.13 | < 0.00001 |  |
| Food x Ecotype | 1 | | 0.15 | 0.15 | 2.60 | 0.11 |  |
| Drainage x Mother | 343 | | 36.70 | 0.11 | 1.83 | < 0.00001 |  |
| Residuals | 315 | | 18.41 | 0.06 |  |  |  |
| Error: within individuals | | |  |  |  |  |  |
| Age | 2 | | 1310.23 | 655.11 | 18348.95 | < 0.00001 |  |
| Age x Food | 2 | | 7.33 | 3.67 | 102.71 | < 0.00001 |  |
| Age x Dataset | 6 | | 29.45 | 4.91 | 137.46 | < 0.00001 |  |
| Age x Food x Dataset | 6 | | 0.70 | 0.12 | 3.26 | 0.00345 |  |
| Residuals | 1330 | | 47.49 | 0.04 |  |  |  |
| Pairwise comparisons: | |  | |  |  |  | |
|  | | Mean ± SD | | Mean ± SD | *F*-value | *p*-value | |
|  | | Group 1 | | Group 2 |  |  | |
| Error: within individuals | |  | |  |  |  | |
| Food [h] Age [1] Data [1-2] | | 45.3 ± 11.6 | | 34.0 ± 7.9 | 91.20 | < 0.00001 | |
| Food [h] Age [1] Data [1-3] | | 45.3 ± 11.6 | | 35.6 ± 7.3 | 60.09 | < 0.00001 | |
| Food [h] Age [1] Data [1-4] | | 45.3 ± 11.6 | | 35.2 ± 7.6 | 75.43 | < 0.00001 | |
| Food [h] Age [1] Data [2-3] | | 34.0 ± 7.9 | | 35.6 ± 7.3 | 4.06 | 0.04530 | |
| Food [h] Age [1] Data [2-4] | | 34.0 ± 7.9 | | 35.2 ± 7.6 | 2.74 | 0.10 | |
| Food [h] Age [1] Data [3-4] | | 35.6 ± 7.3 | | 35.2 ± 7.6 | 0.28 | 0.60 | |
| Food [h] Age [2] Data [1-2] | | 136.2 ± 24.7 | | 156.9 ± 42.6 | 16.59 | < 0.00001 | |
| Food [h] Age [2] Data [1-3] | | 136.2 ± 24.7 | | 231.7 ± 68.8 | 289.40 | < 0.00001 | |
| Food [h] Age [2] Data [1-4] | | 136.2 ± 24.7 | | 195.0 ± 54.4 | 146.90 | < 0.00001 | |
| Food [h] Age [2] Data [2-3] | | 156.9 ± 42.6 | | 231.7 ± 68.8 | 244.90 | < 0.00001 | |
| Food [h] Age [2] Data [2-4] | | 156.9 ± 42.6 | | 195.0 ± 54.4 | 90.69 | < 0.00001 | |
| Food [h] Age [2] Data [3-4] | | 231.7 ± 68.8 | | 195.0 ± 54.4 | 59.35 | < 0.00001 | |
| Food [h] Age [3] Data [1-2] | | 196.0 ± 38.6 | | 218.7 ± 59.5 | 10.01 | 0.00194 | |
| Food [h] Age [3] Data [1-3] | | 196.0 ± 38.6 | | 348.0 ± 83.9 | 335.5 | < 0.00001 | |
| Food [h] Age [3] Data [1-4] | | 196.0 ± 38.6 | | 278.6 ± 62.0 | 139.20 | < 0.00001 | |
| Food [h] Age [3] Data [2-3] | | 218.7 ± 59.5 | | 348.0 ± 83.9 | 331.20 | < 0.00001 | |
| Food [h] Age [3] Data [2-4] | | 218.7 ± 59.5 | | 278.6 ± 62.0 | 109.10 | < 0.00001 | |
| Food [h] Age [3] Data [3-4] | | 348.0 ± 83.9 | | 278.6 ± 62.0 | 81.24 | < 0.00001 | |
| Food [l] Age [1] Data [1-2] | | 44.9 ± 10.9 | | 34.0 ± 7.7 | 66.70 | < 0.00001 | |
| Food [l] Age [1] Data [1-3] | | 44.9 ± 10.9 | | 35.7 ± 8.0 | 47.32 | < 0.00001 | |
| Food [l] Age [1] Data [1-4] | | 44.9 ± 10.9 | | 33.7 ± 7.2 | 88.80 | < 0.00001 | |
| Food [l] Age [1] Data [2-3] | | 34.0 ± 7.7 | | 35.7 ± 8.0 | 2.89 | 0.09 | |
| Food [l] Age [1] Data [2-4] | | 34.0 ± 7.7 | | 33.7 ± 7.2 | 0.05 | 0.83 | |
| Food [l] Age [1] Data [3-4] | | 35.7 ± 8.0 | | 33.7 ± 7.2 | 4.73 | 0.03090 | |
| Food [l] Age [2] Data [1-2] | | 97.6 ± 23.1 | | 124.7 ± 43.5 | 43.29 | < 0.00001 | |
| Food [l] Age [2] Data [1-3] | | 97.6 ± 23.1 | | 192.5 ± 56.1 | 430.30 | < 0.00001 | |
| Food [l] Age [2] Data [1-4] | | 97.6 ± 23.1 | | 139.8 ± 37.6 | 148.6 | < 0.00001 | |
| Food [l] Age [2] Data [2-3] | | 124.7 ± 43.5 | | 192.5 ± 56.1 | 277.9 | < 0.00001 | |
| Food [l] Age [2] Data [2-4] | | 124.7 ± 43.5 | | 139.8 ± 37.6 | 30.89 | < 0.00001 | |
| Food [l] Age [2] Data [3-4] | | 192.5 ± 56.1 | | 139.8 ± 37.6 | 185.00 | < 0.00001 | |
| Food [l] Age [3] Data [1-2] | | 145.9 ± 30.5 | | 180.2 ± 54.2 | 27.08 | < 0.00001 | |
| Food [l] Age [3] Data [1-3] | | 145.9 ± 30.5 | | 292.1 ± 68.1 | 389.40 | < 0.00001 | |
| Food [l] Age [3] Data [1-4] | | 145.9 ± 30.5 | | 185.8 ± 44.2 | 56.64 | < 0.00001 | |
| Food [l] Age [3] Data [2-3] | | 180.2 ± 54.2 | | 292.1 ± 68.1 | 275.90 | < 0.00001 | |
| Food [l] Age [3] Data [2-4] | | 180.2 ± 54.2 | | 185.8 ± 44.2 | 2.89 | 0.09 | |
| Food [l] Age [3] Data [3-4] | | 292.1 ± 68.1 | | 185.8 ± 44.2 | 312.5 | < 0.00001 | |

The analysis includes female wet weights measured at the beginning of the experiment (wt0), at the birth of litters 1 (wt1) and 2 (wt2), but not weights measured at the birth of litter 3 (wt3) because those were lacking from one of the four datasets. We also excluded individuals with missing values. In total, the model included 2019 observations, 673 experimental individuals, 353 mothers of experimental individuals, and seven drainages. Wet weights were measured in milligrams and were base-*e* log-transformed before fitting the model. However, group-specific means and standard deviations for pairwise comparisons are provided as untransformed values for a better intuitive understanding of the direction and magnitude of differences between datasets. Df: degrees of freedom, Sum Sq: sum of squares, Mean Sq: mean squares, SD: standard deviation, h: high, l: low, Data: dataset.

## Table S3. Analysis of variance with repeated measures of female growth until the birth of litter 3 (wt0-wt3).

|  | Df | | Sum Sq | Mean Sq | *F*-value | *p*-value |  |
| --- | --- | --- | --- | --- | --- | --- | --- |
| Error: between individuals | | |  |  |  |  |  |
| Food | 1 | | 25.74 | 25.74 | 302.26 | < 0.00001 |  |
| Dataset | 2 | | 48.77 | 24.39 | 286.40 | < 0.00001 |  |
| Ecotype | 1 | | 1.65 | 1.65 | 19.35 | 0.00002 |  |
| Drainage | 5 | | 12.64 | 2.53 | 29.69 | < 0.00001 |  |
| Food x Dataset | 2 | | 3.71 | 1.85 | 21.77 | < 0.00001 |  |
| Food x Ecotype | 1 | | 0.01 | 0.01 | 0.14 | 0.71 |  |
| Drainage x Mother | 298 | | 42.37 | 0.14 | 1.67 | 0.00001 |  |
| Residuals | 264 | | 22.48 | 0.09 |  |  |  |
| Error: within individuals | | |  |  |  |  |  |
| Age | 3 | | 1688.11 | 562.70 | 20519.21 | < 0.00001 |  |
| Age x Food | 3 | | 7.17 | 2.39 | 87.10 | < 0.00001 |  |
| Age x Dataset | 6 | | 14.40 | 2.40 | 87.51 | < 0.00001 |  |
| Age x Food x Dataset | 6 | | 1.30 | 0.22 | 7.92 | < 0.00001 |  |
| Residuals | 1707 | | 46.81 | 0.03 |  |  |  |
| Pairwise comparisons: | |  | |  |  |  | |
|  | | Mean ± SD | | Mean ± SD |  |  | |
|  | | Group 1 | | Group 2 | *F*-value | p-value | |
| Error: within individuals | |  | |  |  |  | |
| Food [h] Age [1] Data [2-3] | | 34.1 ± 7.9 | | 35.6 ± 7.3 | 3.60 | 0.06 | |
| Food [h] Age [1] Data [2-4] | | 34.1 ± 7.9 | | 35.3 ± 7.5 | 3.05 | 0.08 | |
| Food [h] Age [1] Data [3-4] | | 35.6 ± 7.3 | | 35.3 ± 7.5 | 0.12 | 0.73 | |
| Food [h] Age [2] Data [2-3] | | 156.5 ± 42.7 | | 231.4 ± 69.3 | 226.90 | < 0.00001 | |
| Food [h] Age [2] Data [2-4] | | 156.5 ± 42.7 | | 196.1 ± 54.4 | 97.34 | < 0.00001 | |
| Food [h] Age [2] Data [3-4] | | 231.4 ± 69.3 | | 196.1 ± 54.4 | 53.02 | < 0.00001 | |
| Food [h] Age [3] Data [2-3] | | 218.9 ± 59.8 | | 346.8 ± 83.7 | 336.10 | < 0.00001 | |
| Food [h] Age [3] Data [2-4] | | 218.9 ± 59.8 | | 280.2 ± 61.4 | 119.40 | < 0.00001 | |
| Food [h] Age [3] Data [3-4] | | 346.8 ± 83.7 | | 280.2 ± 61.4 | 75.66 | < 0.00001 | |
| Food [h] Age [4] Data [2-3] | | 294.7 ± 76.5 | | 470.9 ± 102.5 | 301.30 | < 0.00001 | |
| Food [h] Age [4] Data [2-4] | | 294.7 ± 76.5 | | 357.7 ± 77.6 | 64.94 | < 0.00001 | |
| Food [h] Age [4] Data [3-4] | | 470.9 ± 102.5 | | 357.7 ± 77.6 | 118.30 | < 0.00001 | |
| Food [l] Age [1] Data [2-3] | | 34.0 ± 7.9 | | 35.8 ± 8.1 | 3.15 | 0.08 | |
| Food [l] Age [1] Data [2-4] | | 34.0 ± 7.9 | | 33.6 ± 7.2 | 0.13 | 0.72 | |
| Food [l] Age [1] Data [3-4] | | 35.8 ± 8.1 | | 33.6 ± 7.2 | 3.97 | 0.01604 | |
| Food [l] Age [2] Data [2-3] | | 123.8 ± 43.6 | | 190.4 ± 54.9 | 268.20 | < 0.00001 | |
| Food [l] Age [2] Data [2-4] | | 123.8 ± 43.6 | | 140.4 ± 37.3 | 35.27 | < 0.00001 | |
| Food [l] Age [2] Data [3-4] | | 190.4 ± 54.9 | | 140.4 ± 37.3 | 177.5 | < 0.00001 | |
| Food [l] Age [3] Data [2-3] | | 178.7 ± 53.9 | | 290.6 ± 68.1 | 294.95 | < 0.00001 | |
| Food [l] Age [3] Data [2-4] | | 178.7 ± 53.9 | | 186.3 ± 44.1 | 4.70 | 0.03140 | |
| Food [l] Age [3] Data [3-4] | | 290.6 ± 68.1 | | 186.3 ± 44.1 | 314.80 | < 0.00001 | |
| Food [l] Age [4] Data [2-3] | | 242.6 ± 64.2 | | 402.4 ± 84.3 | 281.50 | < 0.00001 | |
| Food [l] Age [4] Data [2-4] | | 242.6 ± 64.2 | | 223.5 ± 49.7 | 6.91 | 0.00928 | |
| Food [l] Age [4] Data [3-4] | | 402.4 ± 84.3 | | 223.5 ± 49.7 | 517.40 | < 0.00001 | |

The analysis includes female wet weights measured at the beginning of the experiment (wt0), at the birth of litters 1 (wt1), 2 (wt2), and 3 (wt3). As dataset 1 lacked weights measured at the birth of litter 3, dataset 1 was excluded from this analysis. We also excluded individuals with missing values. In total, the model included 2300 observations, 575 experimental individuals, 307 mothers of experimental individuals, and six drainages. Wet weights were measured in milligrams and were base-*e* log-transformed before fitting the model. However, group-specific means and standard deviations for pairwise comparisons are provided as untransformed values for a better intuitive understanding of the direction and magnitude of differences between datasets. Df: degrees of freedom, Sum Sq: sum of squares, Mean Sq: mean squares, SD: standard deviation, h: high, l: low, Data: dataset.

## Table S4. Linear mixed-effects model on female age at the beginning of the experiment (age0).

| Predictor |  |  |  |
| --- | --- | --- | --- |
| Fixed effects: | Estimate (S.E.) | *t-*value | *p*-value |
| Intercept | 23.43 (0.67) | 35.16 | 4.04e-09 |
| Food (high vs. low) | -0.00 (0.22) | -0.01 | 1.00 |
| Ecotype (fast vs. slow) | -0.87 (0.24) | -3.68 | 0.00025 |
| Dataset (2 vs. 1) | 4.08 (1.60) | 2.55 | 0.05 |
| Dataset (2 vs. 3) | 2.77 (0.39) | 7.15 | 2.85e-12 |
| Dataset (2 vs. 4) | 7.69 (0.41) | 18.63 | 2.09e-60 |
| Random effects: | Var (S.D.) | $\chi_{1}^{2}$ | *p*-value |
| Drainage | 2.055 (1.434) | 55.54 | 9.17e-14 |
| Residual | 8.448 (2.907) |  |  |

The female age at the beginning of the experiment was measured in days and was fitted as untransformed values. The total number of observations included in the model is 707. Maternal identity could not be included as a random effect, as fish from the same mother came from a single litter, and thus were of the same age. S.E.: standard error, Var: variance, S.D.: standard deviation.

## Table S5. Linear mixed-effects model on male age at the beginning of the experiment (age0m).

| Predictor |  |  |  |
| --- | --- | --- | --- |
| Fixed effects: | Estimate (S.E.) | *t-*value | *p*-value |
| Intercept | 23.56 (0.38) | 61.78 | 2.70e-12 |
| Food (high vs. low) | -0.05 (0.24) | -0.23 | 0.82 |
| Ecotype (fast vs. slow) | -0.06 (0.26) | -0.22 | 0.83 |
| Dataset (2 vs. 1) | 3.53 (0.69) | 5.09 | 0.01194 |
| Dataset (2 vs. 3) | 2.92 (0.32) | 9.17 | 8.59e-15 |
| Random effects: | Var (S.D.) | $\chi_{1}^{2}$ | *p*-value |
| Drainage | 0.297 (0.545) | 2.51 | 0.11 |
| Residual | 6.460 (2.542) |  |  |

The male age at the beginning of the experiment was measured in days and was fitted as untransformed values. The total number of observations included in the model is 459. Maternal identity could not be included as a random effect, as fish from the same mother came from a single litter, and thus were of the same age. S.E.: standard error, Var: variance, S.D.: standard deviation.

## Table S6. Linear mixed-effects model on male age at sexual maturity (agemat).

| Predictor |  |  |  |
| --- | --- | --- | --- |
| Fixed effects: | Estimate (S.E.) | *t-*value | *p*-value |
| Intercept | 3.84 (0.04) | 95.67 | 4.32e-11 |
| Food (high vs. low) | 0.11 (0.01) | 9.40 | 6.98e-18 |
| Ecotype (fast vs. slow) | 0.14 (0.02) | 7.11 | 1.53e-11 |
| Dataset (2 vs. 1) | 0.20 (0.09) | 2.27 | 0.09 |
| Dataset (2 vs. 3) | 0.07 (0.02) | 2.76 | 0.00625 |
| Random effects: | Var (S.D.) | $\chi_{1}^{2}$ | *p*-value |
| Maternal identity | 0.009 (0.095) | 28.70 | 8.47e-08 |
| Drainage | 0.006 (0.075) | 28.31 | 1.03e-07 |
| Residual | 0.016 (0.127) |  |  |

The male age at sexual maturity was measured in days and was base-*e* log-transformed before fitting the model. Model results are provided on the transformed scale. The total number of observations included in the model is 442. S.E.: standard error, Var: variance, S.D.: standard deviation.

## Table S7. Linear mixed-effects model on female age at first birth (agepart1).

| Predictor |  |  |  |
| --- | --- | --- | --- |
| Fixed effects: | Estimate (S.E.) | *t-*value | *p*-value |
| Intercept | 4.37 (0.02) | 194.07 | 2.22e-24 |
| Food (high vs. low) | 0.15 (0.01) | 12.25 | 6.03e-29 |
| Ecotype (fast vs. slow) | 0.08 (0.01) | 6.31 | 9.14e-10 |
| Dataset (2 vs. 1) | 0.04 (0.04) | 0.94 | 0.39 |
| Dataset (2 vs. 3) | -0.04 (0.02) | -2.03 | 0.04387 |
| Dataset (2 vs. 4) | 0.01 (0.02) | 0.24 | 0.81 |
| Random effects: | Var (S.D.) | $\chi_{1}^{2}$ | *p*-value |
| Maternal identity | 0.001 (0.029) | 0.35 | 0.56 |
| Drainage | 0.001 (0.036) | 11.59 | 0.00066 |
| Residual | 0.024 (0.156) |  |  |

The female age at first birth was measured in days and was base-*e* log-transformed before fitting the model. Model results are provided on the transformed scale. The total number of observations included in the model is 687. S.E.: standard error, Var: variance, S.D.: standard deviation.

## Table S8. Linear mixed-effects model on female age at second birth (agepart2).

| Predictor |  |  |  |
| --- | --- | --- | --- |
| Fixed effects: | Estimate (S.E.) | *t-*value | *p*-value |
| Intercept | 4.62 (0.02) | 233.91 | 5.59e-22 |
| Food (high vs. low) | 0.13 (0.01) | 12.44 | 1.43e-29 |
| Ecotype (fast vs. slow) | 0.08 (0.01) | 7.18 | 5.12e-12 |
| Dataset (2 vs. 1) | 0.04 (0.04) | 1.11 | 0.32 |
| Dataset (2 vs. 3) | -0.03 (0.02) | -1.74 | 0.08 |
| Dataset (2 vs. 4) | 0.03 (0.02) | 1.82 | 0.07 |
| Random effects: | Var (S.D.) | $\chi_{1}^{2}$ | *p*-value |
| Maternal identity | 0.000 (0.022) | 0.22 | 0.64 |
| Drainage | 0.001 (0.033) | 9.72 | 0.00182 |
| Residual | 0.017 (0.132) |  |  |

The female age at second birth was measured in days and was base-*e* log-transformed before fitting the model. Model results are provided on the transformed scale. The total number of observations included in the model is 681. S.E.: standard error, Var: variance, S.D.: standard deviation.

## Table S9. Linear mixed-effects model on female age at third birth (agepart3).

| Predictor |  |  |  |
| --- | --- | --- | --- |
| Fixed effects: | Estimate (S.E.) | *t-*value | *p*-value |
| Intercept | 4.82 (0.02) | 244.22 | 5.29e-20 |
| Food (high vs. low) | 0.08 (0.01) | 9.36 | 2.11e-18 |
| Ecotype (fast vs. slow) | 0.09 (0.01) | 7.73 | 1.97e-13 |
| Dataset (2 vs. 3) | -0.01 (0.02) | -0.59 | 0.56 |
| Dataset (2 vs. 4) | 0.05 (0.02) | 2.80 | 0.00596 |
| Random effects: | Var (S.D.) | $\chi_{1}^{2}$ | *p*-value |
| Maternal identity | 0.003 (0.050) | 8.15 | 0.00430 |
| Drainage | 0.001 (0.034) | 9.54 | 0.00202 |
| Residual | 0.012 (0.109) |  |  |

The female age at third birth was measured in days and was base-*e* log-transformed before fitting the model. Model results are provided on the transformed scale. The total number of observations included in the model is 583. S.E.: standard error, Var: variance, S.D.: standard deviation.

## Table S10. Linear mixed-effects model on the percentage fat in a female’s total tissues (fat).

| Predictor |  |  |  |
| --- | --- | --- | --- |
| Fixed effects: | Estimate (S.E.) | *t-*value | *p*-value |
| Intercept | 0.20 (0.01) | 31.52 | 9.18e-76 |
| Food (high vs. low) | -0.02 (0.00) | -3.83 | 0.00019 |
| Ecotype (fast vs. slow) | -0.00 (0.01) | -0.05 | 0.96 |
| Dataset (2 vs. 1) | -0.07 (0.01) | -10.67 | 1.21e-19 |
| Drainage (Madamas vs. Mar.) | 0.01 (0.01) | 1.40 | 0.16 |
| Drainage (Madamas vs. Yarra) | 0.00 (0.01) | 0.56 | 0.58 |
| Random effects: | Var (S.D.) | $\chi_{1}^{2}$ | *p*-value |
| Maternal identity | 0.000 (0.008) | 0.11 | 0.74 |
| Residual | 0.002 (0.040) |  |  |

The percentage fat in a female’s total tissues is a proportion and fitted as untransformed values. The total number of observations included in the model is 260. As the analysis included data from only four drainages, drainage was fitted as a fixed effect. In dataset 1 fish originated from a single drainage (El Cedro), which was not sampled for any other dataset, so effects of dataset 1 and of the El Cedro drainage cannot be disentangled. S.E.: standard error, Mar: Marianne drainage, Var: variance, S.D.: standard deviation.

## Table S11. Linear mixed-effects model on the inter-birth interval 1 (intrvl1).

| Predictor |  |  |  |
| --- | --- | --- | --- |
| Fixed effects: | Estimate (S.E.) | *t-*value | *p*-value |
| Intercept | 3.09 (0.02) | 138.85 | 1.23e-16 |
| Food (high vs. low) | 0.04 (0.01) | 3.49 | 0.00054 |
| Ecotype (fast vs. slow) | 0.07 (0.01) | 5.18 | 4.33e-07 |
| Dataset (2 vs. 1) | 0.07 (0.04) | 1.61 | 0.20 |
| Dataset (2 vs. 3) | 0.03 (0.02) | 1.61 | 0.11 |
| Dataset (2 vs. 4) | 0.15 (0.02) | 6.42 | 7.81e-08 |
| Random effects: | Var (S.D.) | $\chi_{1}^{2}$ | *p*-value |
| Maternal identity | 0.003 (0.051) | 3.20 | 0.07 |
| Drainage | 0.001 (0.032) | 0.24 | 0.63 |
| Residual | 0.025 (0.157) |  |  |

The interval between a female’s birth 1 and 2 was measured in days and was base-*e* log-transformed before fitting the model. Model results are provided on the transformed scale. The total number of observations included in the model is 680. S.E.: standard error, Var: variance, S.D.: standard deviation.

## Table S12. Linear mixed-effects model on the inter-birth interval 2 (intrvl2).

| Predictor |  |  |  |
| --- | --- | --- | --- |
| Fixed effects: | Estimate (S.E.) | *t-*value | *p*-value |
| Intercept | 3.08 (0.03) | 120.42 | 3.59e-16 |
| Food (high vs. low) | 0.02 (0.01) | 2.87 | 0.00442 |
| Ecotype (fast vs. slow) | 0.10 (0.02) | 6.31 | 1.20e-09 |
| Dataset (2 vs. 3) | 0.06 (0.02) | 2.55 | 0.01184 |
| Dataset (2 vs. 4) | 0.10 (0.02) | 4.04 | 0.00010 |
| Random effects: | Var (S.D.) | $\chi_{1}^{2}$ | *p*-value |
| Maternal identity | 0.010 (0.101) | 63.25 | 1.82e-15 |
| Drainage | 0.002 (0.044) | 6.90 | 0.00864 |
| Residual | 0.010 (0.099) |  |  |

The interval between a female’s birth 2 and 3 was measured in days and was base-*e* log-transformed before fitting the model. Model results are provided on the transformed scale. The total number of observations included in the model is 582. S.E.: standard error, Var: variance, S.D.: standard deviation.

## Table S13. Linear mixed-effects model on female standard length at birth 1 (len1).

| Predictor |  |  |  |
| --- | --- | --- | --- |
| Fixed effects: | Estimate (S.E.) | *t-*value | *p*-value |
| Intercept | 19.20 (0.27) | 70.05 | 6.74e-13 |
| Food (high vs. low) | -1.48 (0.11) | -13.59 | 4.53e-34 |
| Ecotype (fast vs. slow) | 0.25 (0.13) | 1.97 | 0.04930 |
| Dataset (2 vs. 1) | -0.89 (0.61) | -1.46 | 0.21 |
| Dataset (2 vs. 3) | 2.56 (0.21) | 12.35 | 3.26e-27 |
| Dataset (2 vs. 4) | 0.37 (0.22) | 1.65 | 0.10 |
| Random effects: | Var (S.D.) | $\chi_{1}^{2}$ | *p*-value |
| Maternal identity | 0.209 (0.458) | 2.82 | 0.09 |
| Drainage | 0.277 (0.526) | 23.06 | 1.57e-06 |
| Residual | 2.029 (1.425) |  |  |

The female standard length at birth 1 was measured in millimetres and was fitted as untransformed values. The total number of observations included in the model is 687. S.E.: standard error, Var: variance, S.D.: standard deviation.

## Table S14. Linear mixed-effects model on female standard length at birth 2 (len2).

| Predictor |  |  |  |
| --- | --- | --- | --- |
| Fixed effects: | Estimate (S.E.) | *t-*value | *p*-value |
| Intercept | 3.07 (0.01) | 211.25 | 1.03e-14 |
| Food (high vs. low) | -0.09 (0.00) | -17.48 | 3.06e-49 |
| Ecotype (fast vs. slow) | 0.01 (0.01) | 0.92 | 0.36 |
| Dataset (2 vs. 1) | -0.05 (0.03) | -1.38 | 0.23 |
| Dataset (2 vs. 3) | 0.15 (0.01) | 16.54 | 4.59e-44 |
| Dataset (2 vs. 4) | 0.00 (0.01) | 0.00 | 1.00 |
| Random effects: | Var (S.D.) | $\chi_{1}^{2}$ | *p*-value |
| Maternal identity | 0.000 (0.014) | 0.55 | 0.46 |
| Drainage | 0.001 (0.030) | 35.28 | 2.85e-09 |
| Residual | 0.004 (0.065) |  |  |

The female standard length at birth 2 was measured in millimetres and was base-*e* log-transformed before fitting the model. Model results are provided on the transformed scale. The total number of observations included in the model is 678. S.E.: standard error, Var: variance, S.D.: standard deviation.

## Table S15. Linear mixed-effects model on female standard length at birth 3 (len3).

| Predictor |  |  |  |
| --- | --- | --- | --- |
| Fixed effects: | Estimate (S.E.) | *t-*value | *p*-value |
| Intercept | 4.89 (0.03) | 141.73 | 6.52e-14 |
| Food (high vs. low) | -0.22 (0.01) | -16.89 | 2.55e-45 |
| Ecotype (fast vs. slow) | 0.01 (0.01) | 0.42 | 0.67 |
| Dataset (2 vs. 3) | 0.37 (0.02) | 17.12 | 4.68e-45 |
| Dataset (2 vs. 4) | -0.08 (0.02) | -3.63 | 0.00034 |
| Random effects: | Var (S.D.) | $\chi_{1}^{2}$ | *p*-value |
| Maternal identity | 0.000 (0.012) | 0.00 | 0.96 |
| Drainage | 0.005 (0.072) | 40.58 | 1.89e-10 |
| Residual | 0.024 (0.154) |  |  |

The female standard length at birth 3 was measured in millimetres and was square-root-transformed before fitting the model. Model results are provided on the transformed scale. The total number of observations included in the model is 583. S.E.: standard error, Var: variance, S.D.: standard deviation.

## Table S16. Linear mixed-effects model on male standard length at sexual maturity (lenmat).

| Predictor |  |  |  |
| --- | --- | --- | --- |
| Fixed effects: | Estimate (S.E.) | *t-*value | *p*-value |
| Intercept | 2.64 (0.01) | 251.73 | 4.23e-18 |
| Food (high vs. low) | -0.05 (0.00) | -10.02 | 5.41e-19 |
| Ecotype (fast vs. slow) | 0.02 (0.01) | 1.87 | 0.06 |
| Dataset (2 vs. 3) | 0.13 (0.01) | 14.81 | 1.01e-23 |
| Random effects: | Var (S.D.) | $\chi_{1}^{2}$ | *p*-value |
| Maternal identity | 0.002 (0.039) | 31.54 | 1.96e-08 |
| Drainage | 0.000 (0.014) | 3.49 | 0.06 |
| Residual | 0.002 (0.046) |  |  |

The male standard length at sexual maturity was measured in millimetres and was base-*e* log-transformed before fitting the model. Model results are provided on the transformed scale. The total number of observations included in the model is 357. S.E.: standard error, Var: variance, S.D.: standard deviation.

## Table S17. Linear mixed-effects model on the mean dry weight of new-born offspring in litter 1 (mnemb1).

| Predictor |  |  |  |
| --- | --- | --- | --- |
| Fixed effects: | Estimate (S.E.) | *t-*value | *p*-value |
| Intercept | 0.82 (0.08) | 10.34 | 0.00125 |
| Food (high vs. low) | 0.07 (0.02) | 4.19 | 3.81e-05 |
| Ecotype (fast vs. slow) | 0.19 (0.02) | 9.07 | 3.36e-17 |
| Dataset (2 vs. 1) | -0.06 (0.17) | -0.37 | 0.74 |
| Dataset (2 vs. 4) | 0.00 (0.04) | -0.02 | 0.99 |
| Random effects: | Var (S.D.) | $\chi_{1}^{2}$ | *p*-value |
| Maternal identity | 0.011 (0.103) | 14.94 | 0.00011 |
| Drainage | 0.023 (0.151) | 63.40 | 1.68e-15 |
| Residual | 0.032 (0.180) |  |  |

The mean dry weight of new-born offspring in litter 1 was measured in milligrams and was fitted as untransformed values. The total number of observations included in the model is 501. S.E.: standard error, Var: variance, S.D.: standard deviation.

## **Table S18. Linear mixed-effects model on the mean dry weight of new-born offspring in litter 2 (mnemb2**).

| Predictor |  |  |  |
| --- | --- | --- | --- |
| Fixed effects: | Estimate (S.E.) | *t-*value | *p*-value |
| Intercept | 0.82 (0.07) | 11.01 | 0.00084 |
| Food (high vs. low) | 0.06 (0.02) | 3.72 | 0.00025 |
| Ecotype (fast vs. slow) | 0.21 (0.02) | 9.73 | 3.84e-19 |
| Dataset (2 vs. 1) | -0.06 (0.16) | -0.40 | 0.72 |
| Dataset (2 vs. 4) | 0.11 (0.04) | 3.00 | 0.00299 |
| Random effects: | Var (S.D.) | $\chi_{1}^{2}$ | *p*-value |
| Maternal identity | 0.015 (0.123) | 29.96 | 4.40e-08 |
| Drainage | 0.020 (0.141) | 61.55 | 4.31e-15 |
| Residual | 0.028 (0.166) |  |  |

The mean dry weight of new-born offspring in litter 2 was measured in milligrams and was fitted as untransformed values. The total number of observations included in the model is 494. S.E.: standard error, Var: variance, S.D.: standard deviation.

## Table S19. Linear mixed-effects model on the mean dry weight of new-born offspring in litter 3 (mnemb3).

| Predictor |  |  |  |
| --- | --- | --- | --- |
| Fixed effects: | Estimate (S.E.) | *t-*value | *p*-value |
| Intercept | 0.82 (0.04) | 21.97 | 3.10e-60 |
| Food (high vs. low) | 0.05 (0.03) | 1.83 | 0.07 |
| Ecotype (fast vs. slow) | 0.25 (0.03) | 9.33 | 2.11e-17 |
| Dataset (2 vs. 4) | 0.10 (0.04) | 2.23 | 0.02704 |
| Drainage (Madamas vs. Mar.) | -0.12 (0.05) | -2.43 | 0.01592 |
| Drainage (Madamas vs. Oro.) | 0.13 (0.06) | 2.21 | 0.02838 |
| Drainage (Madamas vs. Yarra) | -0.09 (0.05) | -1.90 | 0.06 |
| Random effects: | Var (S.D.) | $\chi_{1}^{2}$ | *p*-value |
| Maternal identity | 0.002 (0.047) | 0.07 | 0.79 |
| Residual | 0.065 (0.255) |  |  |

The mean dry weight of new-born offspring in litter 3 was measured in milligrams and was fitted as untransformed values. The total number of observations included in the model is 398. As the analysis included data from only four drainages, drainage was fitted as a fixed effect. S.E.: standard error, Mar: Marianne drainage, Oro: Oropuche drainage, Var: variance, S.D.: standard deviation.

## Table S20. Linear mixed-effects model on the mean percentage fat in new-born offspring in litter 1 (mnembfat1).

| Predictor |  |  |  |
| --- | --- | --- | --- |
| Fixed effects: | Estimate (S.E.) | *t-*value | *p*-value |
| Intercept | 0.25 (0.03) | 9.80 | 0.00080 |
| Food (high vs. low) | 0.00 (0.01) | 0.46 | 0.64 |
| Ecotype (fast vs. slow) | -0.01 (0.01) | -1.40 | 0.16 |
| Dataset (2 vs. 1) | -0.04 (0.05) | -0.74 | 0.52 |
| Dataset (2 vs. 4) | -0.06 (0.02) | -3.15 | 0.00200 |
| Random effects: | Var (S.D.) | $\chi_{1}^{2}$ | *p*-value |
| Maternal identity | 0.003 (0.053) | 18.77 | 1.47e-05 |
| Drainage | 0.002 (0.045) | 14.90 | 0.00011 |
| Residual | 0.007 (0.086) |  |  |

The mean percentage fat in new-born offspring in litter 1 is a proportion and was fitted as untransformed values. The total number of observations included in the model is 497. S.E.: standard error, Var: variance, S.D.: standard deviation.

## Table S21. Linear mixed-effects model on the mean percentage fat in new-born offspring in litter 2 (mnembfat2).

| Predictor |  |  |  |
| --- | --- | --- | --- |
| Fixed effects: | Estimate (S.E.) | *t-*value | *p*-value |
| Intercept | 0.52 (0.03) | 18.14 | 0.00014 |
| Food (high vs. low) | -0.02 (0.01) | -3.25 | 0.00133 |
| Ecotype (fast vs. slow) | -0.00 (0.01) | -0.34 | 0.73 |
| Dataset (2 vs. 1) | -0.03 (0.06) | -0.42 | 0.71 |
| Dataset (2 vs. 4) | -0.09 (0.02) | -5.24 | 4.43e-07 |
| Random effects: | Var (S.D.) | $\chi_{1}^{2}$ | *p*-value |
| Maternal identity | 0.003 (0.059) | 38.90 | 4.46e-10 |
| Drainage | 0.003 (0.053) | 24.43 | 7.70e-07 |
| Residual | 0.005 (0.070) |  |  |

The mean percentage fat in new-born offspring in litter 2 is a proportion and was square-root transformed before fitting the model. The total number of observations included in the model is 484. S.E.: standard error, Var: variance, S.D.: standard deviation.

## Table S22. Linear mixed-effects model on the mean percentage fat in new-born offspring in litter 3 (mnembfat3).

| Predictor |  |  |  |
| --- | --- | --- | --- |
| Fixed effects: | Estimate (S.E.) | *t-*value | *p*-value |
| Intercept | 0.58 (0.02) | 37.41 | 5.20e-104 |
| Food (high vs. low) | -0.04 (0.01) | -4.17 | 4.68e-05 |
| Ecotype (fast vs. slow) | -0.02 (0.01) | -1.98 | 0.04955 |
| Dataset (2 vs. 4) | -0.14 (0.02) | -7.93 | 1.31e-13 |
| Drainage (Madamas vs. Mar.) | -0.05 (0.02) | -2.65 | 0.00878 |
| Drainage (Madamas vs. Oro.) | 0.03 (0.02) | 1.13 | 0.26 |
| Drainage (Madamas vs. Yarra) | -0.01 (0.02) | -0.65 | 0.52 |
| Random effects: | Var (S.D.) | $\chi_{1}^{2}$ | *p*-value |
| Maternal identity | 0.001 (0.035) | 2.01 | 0.16 |
| Residual | 0.008 (0.091) |  |  |

The mean percentage fat in new-born offspring in litter 3 is a proportion and was square-root-transformed before fitting the model. The total number of observations included in the model is 379. As the analysis included data from only four drainages, drainage was fitted as a fixed effect. S.E.: standard error, Mar: Marianne drainage, Oro: Oropuche drainage, Var: variance, S.D.: standard deviation.

## Table S23. Linear mixed-effects model on the number of offspring in litter 1 (n1).

| Predictor |  |  |  |
| --- | --- | --- | --- |
| Fixed effects: | Estimate (S.E.) | *t-*value | *p*-value |
| Intercept | 1.26 (0.09) | 13.99 | 5.91e-07 |
| Food (high vs. low) | -0.39 (0.05) | -8.19 | 1.33e-15 |
| Ecotype (fast vs. slow) | -0.20 (0.05) | -4.04 | 6.22e-05 |
| Dataset (2 vs. 1) | -0.15 (0.18) | -0.84 | 0.46 |
| Dataset (2 vs. 3) | 0.27 (0.08) | 3.44 | 0.00079 |
| Dataset (2 vs. 4) | -0.11 (0.09) | -1.35 | 0.18 |
| Random effects: | Var (S.D.) | $\chi_{1}^{2}$ | *p*-value |
| Maternal identity | 0.000 (0.000) | 0.00 | 1.00 |
| Drainage | 0.022 (0.150) | 0.34 | 0.56 |
| Residual | 0.382 (0.618) |  |  |

The number of offspring in litter 1 was base-*e* log-transformed before fitting the model. Model results are provided on the transformed scale. The total number of observations included in the model is 687. S.E.: standard error, Var: variance, S.D.: standard deviation.

## Table S24. Linear mixed-effects model on the maternal-weight-adjusted number of offspring in litter 1 (n1_wt1adj).

| Predictor |  |  |  |
| --- | --- | --- | --- |
| Fixed effects: | Estimate (S.E.) | *t-*value | *p*-value |
| Intercept | 1.33 (0.11) | 12.08 | 7.85e-06 |
| Food (high vs. low) | -0.13 (0.05) | -2.85 | 0.00452 |
| Ecotype (fast vs. slow) | -0.28 (0.05) | -5.92 | 7.74e-09 |
| Dataset (2 vs. 1) | -0.02 (0.25) | -0.09 | 0.93 |
| Dataset (2 vs. 3) | -0.21 (0.08) | -2.51 | 0.01274 |
| Dataset (2 vs. 4) | -0.24 (0.08) | -2.91 | 0.00393 |
| Maternal weight | 0.01 (0.00) | 12.81 | 8.85e-34 |
| Random effects: | Var (S.D.) | $\chi_{1}^{2}$ | *p*-value |
| Maternal identity | 0.012 (0.110) | 0.45 | 0.50 |
| Drainage | 0.049 (0.222) | 8.75 | 0.00310 |
| Residual | 0.293 (0.542) |  |  |

The number of offspring in litter 1 was base-*e* log-transformed before fitting the model. Model results are provided on the transformed scale. To account for the contribution of female size to fecundity, we used the mean-centered postpartum maternal wet weight (wt1) as a covariate. The total number of observations included in the model is 684. S.E.: standard error, Var: variance, S.D.: standard deviation.

## Table S25. Linear mixed-effects model on the number of offspring in litter 2 (n2).

| Predictor |  |  |  |
| --- | --- | --- | --- |
| Fixed effects: | Estimate (S.E.) | *t-*value | *p*-value |
| Intercept | 2.78 (0.05) | 53.05 | 2.71e-203 |
| Food (high vs. low) | -0.51 (0.04) | -12.19 | 1.09e-28 |
| Ecotype (fast vs. slow) | -0.18 (0.04) | -4.07 | 5.95e-05 |
| Dataset (2 vs. 1) | 0.09 (0.08) | 1.18 | 0.24 |
| Dataset (2 vs. 3) | 0.91 (0.06) | 15.01 | 1.06e-39 |
| Dataset (2 vs. 4) | -0.21 (0.06) | -3.65 | 0.00030 |
| Random effects: | Var (S.D.) | $\chi_{1}^{2}$ | *p*-value |
| Maternal identity | 0.014 (0.118) | 0.50 | 0.48 |
| Drainage | 0.000 (0.000) | 0.00 | 1.00 |
| Residual | 0.301 (0.548) |  |  |

The number of offspring in litter 2 was square-root-transformed before fitting the model. Model results are provided on the transformed scale. The total number of observations included in the model is 682. S.E.: standard error, Var: variance, S.D.: standard deviation.

## Table S26. Linear mixed-effects model on the maternal-weight-adjusted number of offspring in litter 2 (n2_wt2adj).

| Predictor |  |  |  |
| --- | --- | --- | --- |
| Fixed effects: | Estimate (S.E.) | *t-*value | *p*-value |
| Intercept | 2.89 (0.09) | 31.80 | 1.92e-09 |
| Food (high vs. low) | -0.28 (0.04) | -6.36 | 4.97e-10 |
| Ecotype (fast vs. slow) | -0.25 (0.05) | -5.41 | 1.22e-07 |
| Dataset (2 vs. 1) | 0.16 (0.20) | 0.80 | 0.47 |
| Dataset (2 vs. 3) | 0.33 (0.09) | 3.80 | 0.00018 |
| Dataset (2 vs. 4) | -0.29 (0.08) | -3.64 | 0.00037 |
| Maternal weight | 0.00 (0.00) | 10.87 | 1.98e-25 |
| Random effects: | Var (S.D.) | $\chi_{1}^{2}$ | *p*-value |
| Maternal identity | 0.038 (0.195) | 6.05 | 0.01393 |
| Drainage | 0.028 (0.167) | 8.08 | 0.00447 |
| Residual | 0.231 (0.481) |  |  |

The number of offspring in litter 2 was square-root-transformed before fitting the model. Model results are provided on the transformed scale. To account for the contribution of female size to fecundity, we used the mean-centered postpartum maternal wet weight (wt2) as a covariate. The total number of observations included in the model is 675. S.E.: standard error, Var: variance, S.D.: standard deviation.

## Table S27. Linear mixed-effects model on the number of offspring in litter 3 (n3).

| Predictor |  |  |  |
| --- | --- | --- | --- |
| Fixed effects: | Estimate (S.E.) | *t-*value | *p*-value |
| Intercept | 3.62 (0.10) | 37.57 | 7.36e-11 |
| Food (high vs. low) | -0.67 (0.05) | -13.73 | 1.62e-33 |
| Ecotype (fast vs. slow) | -0.27 (0.06) | -4.77 | 3.03e-06 |
| Dataset (2 vs. 3) | 1.13 (0.08) | 13.47 | 5.25e-26 |
| Dataset (2 vs. 4) | -0.51 (0.09) | -5.67 | 1.50e-07 |
| Random effects: | Var (S.D.) | $\chi_{1}^{2}$ | *p*-value |
| Maternal identity | 0.032 (0.179) | 1.93 | 0.17 |
| Drainage | 0.026 (0.162) | 2.57 | 0.11 |
| Residual | 0.341 (0.584) |  |  |

The number of offspring in litter 3 was square-root-transformed before fitting the model. Model results are provided on the transformed scale. The total number of observations included in the model is 581. S.E.: standard error, Var: variance, S.D.: standard deviation.

## Table S28. Linear mixed-effects model on the maternal-weight-adjusted number of offspring in litter 3 (n3_wt3adj).

| Predictor |  |  |  |
| --- | --- | --- | --- |
| Fixed effects: | Estimate (S.E.) | *t-*value | *p*-value |
| Intercept | 3.68 (0.11) | 33.15 | 1.22e-09 |
| Food (high vs. low) | -0.41 (0.05) | -7.72 | 9.67e-14 |
| Ecotype (fast vs. slow) | -0.31 (0.06) | -5.53 | 7.19e-08 |
| Dataset (2 vs. 3) | 0.60 (0.10) | 6.05 | 4.49e-09 |
| Dataset (2 vs. 4) | -0.53 (0.09) | -5.91 | 1.56e-08 |
| Maternal weight | 0.00 (0.00) | 8.81 | 1.53e-17 |
| Random effects: | Var (S.D.) | $\chi_{1}^{2}$ | *p*-value |
| Maternal identity | 0.045 (0.212) | 4.72 | 0.02987 |
| Drainage | 0.045 (0.213) | 13.74 | 0.00021 |
| Residual | 0.285 (0.534) |  |  |

The number of offspring in litter 3 was square-root-transformed before fitting the model. Model results are provided on the transformed scale. To account for the contribution of female size to fecundity, we used the mean-centered postpartum maternal wet weight (wt3) as a covariate. The total number of observations included in the model is 581. S.E.: standard error, Var: variance, S.D.: standard deviation.

## Table S29. Linear mixed-effects model on the reproductive allotment (repall).

| Predictor |  |  |  |
| --- | --- | --- | --- |
| Fixed effects: | Estimate (S.E.) | *t-*value | *p*-value |
| Intercept | 0.11 (0.01) | 15.02 | 6.77e-37 |
| Food (high vs. low) | -0.00 (0.01) | -0.36 | 0.72 |
| Ecotype (fast vs. slow) | -0.01 (0.01) | -1.17 | 0.24 |
| Dataset (2 vs. 1) | 0.03 (0.01) | 3.65 | 0.00032 |
| Drainage (Madamas vs. Mar.) | 0.01 (0.01) | 1.32 | 0.19 |
| Drainage (Madamas vs. Yarra) | 0.02 (0.01) | 2.47 | 0.01401 |
| Random effects: | Var (S.D.) | $\chi_{1}^{2}$ | *p*-value |
| Maternal identity | 0.000 (0.000) | 0.00 | 1.00 |
| Residual | 0.002 (0.048) |  |  |

The reproductive allotment is a proportion and was fitted as untransformed values. The total number of observations included in the model is 259. As the analysis included data from only four drainages, drainage was fitted as a fixed effect. In dataset 1 fish originated from a single drainage (El Cedro), which was not sampled for any other dataset, so the effects of dataset 1 and of the El Cedro drainage cannot be disentangled. S.E.: standard error, Mar: Marianne drainage, Var: variance, S.D.: standard deviation.

## Table S30. Linear mixed-effects model on the percentage fat in a female’s reproductive tissues (repfat).

| Predictor |  |  |  |
| --- | --- | --- | --- |
| Fixed effects: | Estimate (S.E.) | *t-*value | *p*-value |
| Intercept | -1.18 (0.02) | -47.69 | 1.25e-104 |
| Food (high vs. low) | 0.00 (0.02) | 0.10 | 0.92 |
| Ecotype (fast vs. slow) | -0.01 (0.02) | -0.48 | 0.63 |
| Dataset (2 vs. 1) | -0.10 (0.03) | -3.83 | 0.00020 |
| Drainage (Madamas vs. Mar.) | -0.04 (0.03) | -1.51 | 0.13 |
| Drainage (Madamas vs. Yarra) | 0.02 (0.03) | 0.81 | 0.42 |
| Random effects: | Var (S.D.) | $\chi_{1}^{2}$ | *p*-value |
| Maternal identity | 0.001 (0.025) | 0.01 | 0.91 |
| Residual | 0.025 (0.159) |  |  |

The percentage fat in a female’s reproductive tissues is a proportion and was base-*e* log-transformed before fitting the model. Model results are provided on the transformed scale. The total number of observations included in the model is 255. As the analysis included data from only four drainages, drainage was fitted as a fixed effect. In dataset 1 fish originated from a single drainage (El Cedro), which was not sampled for any other dataset, so the effects of dataset 1 and of the El Cedro drainage cannot be disentangled. S.E.: standard error, Mar: Marianne drainage, Var: variance, S.D.: standard deviation.

## Table S31. Linear mixed-effects model on the dry weight of a female’s reproductive tissues (repwt).

| Predictor |  |  |  |
| --- | --- | --- | --- |
| Fixed effects: | Estimate (S.E.) | *t-*value | *p*-value |
| Intercept | 3.48 (0.12) | 29.86 | 9.92e-71 |
| Food (high vs. low) | -0.52 (0.09) | -6.05 | 1.30e-08 |
| Ecotype (fast vs. slow) | 0.01 (0.10) | 0.15 | 0.88 |
| Dataset (2 vs. 1) | -0.91 (0.13) | -7.09 | 7.15e-11 |
| Drainage (Madamas vs. Mar) | 0.27 (0.14) | 1.91 | 0.06 |
| Drainage (Madamas vs. Yarra) | -0.18 (0.14) | -1.25 | 0.21 |
| Random effects: | Var (S.D.) | $\chi_{1}^{2}$ | *p*-value |
| Maternal identity | 0.072 (0.268) | 1.77 | 0.18 |
| Residual | 0.476 (0.690) |  |  |

The dry weight of a female’s reproductive tissues was measured in milligrams and was square-root-transformed before fitting the model. Model results are provided on the transformed scale. The total number of observations included in the model is 262. As the analysis included data from only four drainages, drainage was fitted as a fixed effect. In dataset 1 fish originated from a single drainage (El Cedro), which was not sampled for any other dataset, so the effects of dataset 1 and the El Cedro drainage cannot be disentangled. S.E.: standard error, Mar: Marianne drainage, Var: variance, S.D.: standard deviation.

## Table S32. Linear mixed-effects model on the percentage fat in a female’s somatic tissues (somfat).

| Predictor |  |  |  |
| --- | --- | --- | --- |
| Fixed effects: | Estimate (S.E.) | *t-*value | *p*-value |
| Intercept | 0.17 (0.01) | 25.99 | 4.37e-62 |
| Food (high vs. low) | -0.02 (0.00) | -4.19 | 4.98e-05 |
| Ecotype (fast vs. slow) | 0.00 (0.01) | 0.32 | 0.75 |
| Dataset (2 vs. 1) | -0.08 (0.01) | -10.37 | 7.05e-19 |
| Drainage (Madamas vs. Mar.) | 0.01 (0.01) | 1.48 | 0.14 |
| Drainage (Madamas vs. Yarra) | 0.00 (0.01) | 0.19 | 0.85 |
| Random effects: | Var (S.D.) | $\chi_{1}^{2}$ | *p*-value |
| Maternal identity | 0.000 (0.016) | 2.30 | 0.13 |
| Residual | 0.002 (0.039) |  |  |

The percentage fat in a female’s somatic tissues is a proportion and was fitted as untransformed values. The total number of observations included in the model is 262. As the analysis included data from only four drainages, drainage was fitted as a fixed effect. In dataset 1 fish originated from a single drainage (El Cedro), which was not sampled for any other dataset, so the effects of dataset 1 and of the El Cedro drainage cannot be disentangled. S.E.: standard error, Mar: Marianne drainage, Var: variance, S.D.: standard deviation.

## Table S33. Linear mixed-effects model on the dry weight of a female’s somatic tissues (somwt).

| Predictor |  |  |  |
| --- | --- | --- | --- |
| Fixed effects: | Estimate (S.E.) | *t-*value | *p*-value |
| Intercept | 4.12 (0.04) | 97.28 | 6.30e-156 |
| Food (high vs. low) | -0.24 (0.03) | -7.45 | 9.05e-12 |
| Ecotype (fast vs. slow) | 0.04 (0.04) | 1.10 | 0.27 |
| Dataset (2 vs. 1) | -0.60 (0.05) | -12.98 | 2.31e-25 |
| Drainage (Madamas vs. Mar.) | -0.09 (0.05) | -1.69 | 0.09 |
| Drainage (Madamas vs. Yarra) | -0.18 (0.05) | -3.49 | 0.00065 |
| Random effects: | Var (S.D.) | $\chi_{1}^{2}$ | *p*-value |
| Maternal identity | 0.008 (0.088) | 1.08 | 0.30 |
| Residual | 0.066 (0.258) |  |  |

The dry weight of a female’s somatic tissues was measured in milligrams and was base-*e* log-transformed before fitting the model. Model results are provided on the transformed scale. The total number of observations included in the model is 263. As the analysis included data from only four drainages, drainage was fitted as a fixed effect. In dataset 1 fish originated from a single drainage (El Cedro), which was not sampled for any other dataset, so the effects of dataset 1 and the El Cedro drainage cannot be disentangled. S.E.: standard error, Mar: Marianne drainage, Var: variance, S.D.: standard deviation.

## Table S34. Linear mixed-effects model on the female wet weight at the beginning of the experiment (wt0).

| Predictor |  |  |  |
| --- | --- | --- | --- |
| Fixed effects: | Estimate (S.E.) | *t-*value | *p*-value |
| Intercept | 3.52 (0.04) | 87.09 | 5.89e-16 |
| Food (high vs. low) | -0.01 (0.01) | -1.81 | 0.07 |
| Ecotype (fast vs. slow) | -0.06 (0.02) | -2.60 | 0.00981 |
| Dataset (2 vs. 1) | 0.30 (0.09) | 3.48 | 0.01749 |
| Dataset (2 vs. 3) | 0.08 (0.04) | 2.21 | 0.02814 |
| Dataset (2 vs. 4) | 0.09 (0.04) | 2.42 | 0.01660 |
| Random effects: | Var (S.D.) | $\chi_{1}^{2}$ | *p*-value |
| Maternal identity | 0.034 (0.184) | 305.13 | 2.52e-68 |
| Drainage | 0.005 (0.072) | 14.97 | 0.00011 |
| Residual | 0.011 (0.103) |  |  |

The female wet weight at the beginning of the experiment was measured in milligrams and was base-*e* log-transformed before fitting the model. Model results are provided on the transformed scale. The total number of observations included in the model is 706. S.E.: standard error, Var: variance, S.D.: standard deviation.

## Table S35. Linear mixed-effects model on the male wet weight at the beginning of the experiment (wt0m).

| Predictor |  |  |  |
| --- | --- | --- | --- |
| Fixed effects: | Estimate (S.E.) | *t-*value | *p*-value |
| Intercept | 3.49 (0.04) | 90.16 | 7.25e-14 |
| Food (high vs. low) | 0.00 (0.01) | 0.49 | 0.63 |
| Ecotype (fast vs. slow) | -0.07 (0.03) | -2.74 | 0.00680 |
| Dataset (2 vs. 1) | 0.24 (0.07) | 3.16 | 0.03293 |
| Dataset (2 vs. 3) | 0.13 (0.03) | 3.97 | 0.00013 |
| Random effects: | Var (S.D.) | $\chi_{1}^{2}$ | *p*-value |
| Maternal identity | 0.032 (0.178) | 267.75 | 3.52e-60 |
| Drainage | 0.004 (0.060) | 5.78 | 0.01622 |
| Residual | 0.006 (0.078) |  |  |

The male wet weight at the beginning of the experiment was measured in milligrams and was base-*e* log-transformed before fitting the model. Model results are provided on the transformed scale. The total number of observations included in the model is 458. S.E.: standard error, Var: variance, S.D.: standard deviation.

## Table S36. Linear mixed-effects model on the female wet weight at birth 1 (wt1).

| Predictor |  |  |  |
| --- | --- | --- | --- |
| Fixed effects: | Estimate (S.E.) | *t-*value | *p*-value |
| Intercept | 4.99 (0.05) | 95.94 | 3.43e-13 |
| Food (high vs. low) | -0.28 (0.02) | -15.79 | 9.18e-43 |
| Ecotype (fast vs. slow) | 0.08 (0.02) | 3.60 | 0.00037 |
| Dataset (2 vs. 1) | -0.16 (0.12) | -1.35 | 0.24 |
| Dataset (2 vs. 3) | 0.47 (0.04) | 13.26 | 1.25e-31 |
| Dataset (2 vs. 4) | 0.15 (0.04) | 3.88 | 0.00013 |
| Random effects: | Var (S.D.) | $\chi_{1}^{2}$ | *p*-value |
| Maternal identity | 0.008 (0.089) | 5.58 | 0.01820 |
| Drainage | 0.011 (0.107) | 50.69 | 1.08e-12 |
| Residual | 0.052 (0.229) |  |  |

The female wet weight at birth 1 was measured in milligrams and was base-*e* log-transformed before fitting the model. Model results are provided on the transformed scale. The total number of observations included in the model is 686. S.E.: standard error, Var: variance, S.D.: standard deviation.

## Table S37. Linear mixed-effects model on the female wet weight at birth 2 (wt2).

| Predictor |  |  |  |
| --- | --- | --- | --- |
| Fixed effects: | Estimate (S.E.) | *t-*value | *p*-value |
| Intercept | 5.37 (0.05) | 109.56 | 5.72e-13 |
| Food (high vs. low) | -0.28 (0.02) | -17.18 | 4.72e-48 |
| Ecotype (fast vs. slow) | 0.06 (0.02) | 2.93 | 0.00360 |
| Dataset (2 vs. 1) | -0.14 (0.12) | -1.24 | 0.27 |
| Dataset (2 vs. 3) | 0.54 (0.03) | 17.17 | 2.20e-46 |
| Dataset (2 vs. 4) | 0.11 (0.03) | 3.23 | 0.00136 |
| Random effects: | Var (S.D.) | $\chi_{1}^{2}$ | *p*-value |
| Maternal identity | 0.004 (0.065) | 2.26 | 0.13 |
| Drainage | 0.010 (0.102) | 51.05 | 9.01e-13 |
| Residual | 0.044 (0.210) |  |  |

The female wet weight at birth 2 was measured in milligrams and was base-*e* log-transformed before fitting the model. Model results are provided on the transformed scale. The total number of observations included in the model is 675. S.E.: standard error, Var: variance, S.D.: standard deviation.

## Table S38. Linear mixed-effects model on the female wet weight at birth 3 (wt3).

| Predictor |  |  |  |
| --- | --- | --- | --- |
| Fixed effects: | Estimate (S.E.) | *t-*value | *p*-value |
| Intercept | 5.69 (0.05) | 122.25 | 5.18e-14 |
| Food (high vs. low) | -0.30 (0.02) | -17.17 | 2.99e-46 |
| Ecotype (fast vs. slow) | 0.05 (0.02) | 2.54 | 0.01165 |
| Dataset (2 vs. 3) | 0.54 (0.03) | 17.90 | 1.97e-47 |
| Dataset (2 vs. 4) | 0.00 (0.03) | 0.11 | 0.91 |
| Random effects: | Var (S.D.) | $\chi_{1}^{2}$ | *p*-value |
| Maternal identity | 0.002 (0.048) | 0.60 | 0.44 |
| Drainage | 0.009 (0.096) | 51.89 | 5.87e-13 |
| Residual | 0.043 (0.207) |  |  |

The female wet weight at birth 3 was measured in milligrams and was base-*e* log-transformed before fitting the model. Model results are provided on the transformed scale. The total number of observations included in the model is 583. S.E.: standard error, Var: variance, S.D.: standard deviation.

## Table S39. Linear mixed-effects model on the male wet weight at sexual maturity (wtmat).

| Predictor |  |  |  |
| --- | --- | --- | --- |
| Fixed effects: | Estimate (S.E.) | *t-*value | *p*-value |
| Intercept | 4.11 (0.04) | 100.65 | 4.63e-14 |
| Food (high vs. low) | -0.21 (0.01) | -14.99 | 1.89e-35 |
| Ecotype (fast vs. slow) | 0.06 (0.02) | 2.61 | 0.00979 |
| Dataset (2 vs. 1) | 0.02 (0.08) | 0.27 | 0.80 |
| Dataset (2 vs. 3) | 0.40 (0.03) | 13.09 | 1.50e-26 |
| Random effects: | Var (S.D.) | $\chi_{1}^{2}$ | *p*-value |
| Maternal identity | 0.017 (0.129) | 45.74 | 1.35e-11 |
| Drainage | 0.005 (0.069) | 18.58 | 1.63e-05 |
| Residual | 0.021 (0.144) |  |  |

The male wet weight at sexual maturity was measured in milligrams and was base-*e* log-transformed before fitting the model. Model results are provided on the transformed scale. The total number of observations included in the model is 443. S.E.: standard error, Var: variance, S.D.: standard deviation.

# Supporting References

R CORE TEAM 2020. R: A language and environment for statistical computing. R Foundation for Statistical Computing, Vienna, Austria. <https://www.R-project.org/>.

REZNICK, D. 1983. The structure of guppy life histories: The tradeoff between growth and reproduction. *Ecology,* 64**,** 862-873.

REZNICK, D., BRYANT, M. & HOLMES, D. 2006. The evolution of senescence and post-reproductive lifespan in guppies (*Poecilia reticulata*). *PLoS Biology,* 4**,** 0136-0143.

REZNICK, D. N. 1980. *Life history evolution in the guppy (Poecilia reticulata). .* Ph.D. dissertation, University of Pennsylvania.

REZNICK, D. N., BRYANT, M. J., ROFF, D., GHALAMBOR, C. K. & GHALAMBOR, D. E. 2004. Effect of extrinsic mortality on the evolution of senescence in guppies. *Nature,* 431**,** 1095-1099.

REZNICK, D. N. & BRYGA, H. 1987. Life-history evolution in guppies (*Poecilia reticulata*): 1. Phenotypic and genetic changes in an introduction experiment. *Evolution,* 41**,** 1370-1385.

REZNICK, D. N. & BRYGA, H. A. 1996. Life-history evolution in guppies (*Poecilia reticulata*: Poeciliidae). V. Genetic basis of parallelism in life histories. *The American Naturalist,* 147**,** 339-359.

REZNICK, D. N. & TRAVIS, J. 2019. Experimental studies of evolution and eco-evo dynamics in guppies (*Poecilia reticulata*). *Annual Review of Ecology, Evolution, and Systematics,* 50**,** 335-354.
